# Supplementary material for: Spatial molecular-dynamically ordered NMR spectroscopy of intact bodies and heterogeneous systems
Source: Commun Chem. 2020 Jun 26;3:80. doi: 10.1038/s42004-020-0330-1 (PMC9814264; doi:10.1038/s42004-020-0330-1)
Supplement: Supplementary file 2 — Supplementary Information [file 42004_2020_330_MOESM2_ESM.pdf]

Spatial molecular-dynamically ordered NMR spectroscopy of intact bodies and heterogeneous systems

Kengo Ito et al.

A. Supplementary Methods

Details of processing pseudo-3D SMOOSY spectra by SMOOSY processor

First, spatial molecular-dynamically ordered spectroscopy (SMOOSY) processor used the python Nmrglue package[1] to read and convert the Bruker binary data into a numeric matrix in developed SMOOSY processor. The Numpy and Scipy packages were used for general scientific calculations, and the Matplotlib and Tkinter packages were called for processing on the graphical user interface. The Levenberg–Marquardt method[2] in the Scipy package was used for optimizing the exponential curve fitting of the unprocessed diffusion or relaxation dimension. The diffusion coefficients or relaxation times (x) were calculated by minimizing the sum of squares of a set of Supplementary Equations:

$$x = \underset{y}{argmin} \sum f(y)^2 \tag{1}$$

with $f(y)$ defined as Supplementary Equation 2[3] for diffusion driven SMOOSY (D-SMOOSY), Supplementary Equation 3 for spin–spin relaxation driven SMOOSY (T_2-SMOOSY) and T_2 relaxation-encoded selective total correlation spectroscopy driven SMOOSY ($REST_2$-SMOOSY), and Supplementary Equation 4 for spin–lattice relaxation driven SMOOSY (T_1-SMOOSY) and $REST_1$ driven SMOOSY ($REST_1$-SMOOSY).

$$f(y) = f(I_0, D) = Expt. - I_0 e^{-\gamma^2 g(t)^2 \delta'^{\,2} (2/\pi)^2 \left(\Delta - \delta'/4\right) D} \tag{2}$$

$$f(y) = f(I_0, T_2) = Expt. - I_0 e^{-\tau(t)/T_2} \tag{3}$$

$$f(y) = f(I_0, a, T_1) = Expt. - I_0 (1 - a e^{-\tau(t)/T_1}) \tag{4}$$

I_0 denotes the signal intensity when $g(t)$ is 0 G/cm or $\tau(t)$ is 0 second; D denotes the diffusion coefficient; T_1 and T_2 denote the spin–lattice relaxation and spin–spin relaxation times, respectively; a denotes a scaling factor; *Expt.* denotes the intensity of the observed signal, which is changed by $g(t)$ or $\tau(t)$; γ denotes the gyromagnetic ratio of the observed nucleus; g denotes the gradient strength changed by the observed point t; Δ denotes the diffusion time; δ' denotes the length of the gradient pulse; and τ denotes the total evolution time changed by the observed point t. In addition, the parameter $\tau(t)$ is the variable delay list (*vdlist*) and $g(t)$ is the absolute gradient strength value list (*difflist*) for relaxation or diffusion experiments using the Bruker instrument. These calculated values are mapped to the chemical shift imaging (CSI) signal position (Supplementary Figure 3c). The field of view (*FOV*)[4] along the

spatial z-position was calculated from the following:

$$FOV = \frac{2\pi N}{2\gamma G_{max} 2\delta} \quad (5)$$

where N denotes the number of slices recorded along spatial z-positon, γ denotes the gyromagnetic ratio of the observed nucleus, G_{max} denotes the maximum strength of the magnetic field gradient, and δ denotes the length of the gradient pulse. The developed SMOOSY processor also included *icoshift*[5] (https://github.com/mfitzp/icoshift) as a signal alignment function. Further details of the SMOOSY processor are shown in Supplementary Figure 3; this tool and program can be downloaded from the website http://dmar.riken.jp/Rscripts/. SMOOSY processor makes it easy to visually and intuitively grasp the diffusion or relaxation of the compounds at each spatial z-position.

Homogenous sample preparation for NMR experiments using HR-MAS

For nuclear magnetic resonance (NMR) experiments by high-resolution magic angle spinning (HR-MAS) using homogeneous gel sample, sucrose and alanine were prepared to a final concentration of 12.5 mM, agarose was prepared to 2% (w/v), and sodium 2,2-dimethyl-2-silapentane-5-sulfonate-d6 (DSS-d6) was prepared to a final concentration of 1 mM in deuterium oxide (D_2O) buffer. This sample was melted at 99°C, and the melted sample was put into the 4 mm HR-MAS rotor. After this, the sample in rotor was cooled at room temperature.

NMR experiments with HR-MAS using homogeneous sample

The spectra of homogeneous sample were recorded using an Avance III HD-500 instrument (Bruker Corp., Billerica, MA) equipped with a triple-resonance 4 mm HR-MAS probe with a z-axis gradient operating at 500.13 MHz for ^{1}H and at 125.76 MHz for ^{13}C. CSI, traditional pseudo-two-dimensional (2D) diffusion-ordered spectroscopy (DOSY), and pseudo-three-dimensional (3D) D-SMOOSY spectra of homogeneous sample were recorded at a MAS frequency of 3 kHz at 299 K. The CSI analysis was performed to calculate scaling factor for normalize the signal intensity at each spatial z-position. The traditional DOSY and D-SMOOSY analysis was performed to evaluate whether imaging gradients on the diffusion measurement affect to calculate the diffusion coefficients. In the CSI experiment, 128 complex F1 (^{1}H) points, 2048 complex F2 (^{1}H) points were recorded from 24 scans per F1 increment. The spectral widths obtained for F2 was 17 ppm. The maximum gradient strength for F1 was 9.63 G/cm, and the minimum gradient strength for F1 was −9.63 G/cm, the 128 increment was equal intervals. The details of experimental parameters are shown in Supplementary Tables 3 and 4. In the traditional DOSY experiment, 16,384 complex F2 (^{1}H) points and 16 points of gradient strength on F1 were recorded from 16 scans per F1 increment. The spectral widths obtained for F2 was 16 ppm. The 16 increments in the diffusion list used for F1 were 0.963, 3.948, 6.934, 9.919, 12.904, 15.89, 18.875,

21.86, 24.845, 27.831, 30.816, 33.801, 36.787, 39.772, 42.757, and 45.742 G/cm. The diffusion time was 60 ms. In the *D*-SMOOSY experiment, 128 complex F1 (^{1}H) points, 2048 complex F3 (^{1}H) points, and 24 points of gradient strength on F2 were recorded from 16 scans per F1 and F2 increment. The spectral widths obtained for F3 was 16 ppm. The details of experimental parameters are shown in Supplementary Tables 3. 2D CSI and pseudo-3D *D*-SMOOSY spectra were processed by SMOOSY processor. The exported csv file including a matrix of signal intensities of 2D CSI spectrum was used for calculate the scaling factors at each spatial *z*-position. Also, the exported csv file including a matrix of diffusion coefficients of pseudo-2D *D*-SMOOSY spectral image was used for whether the diffusion coefficients were changed at each spatial *z*-position. To compare between traditional pseudo-2D DOSY spectrum and sliced pseudo-2D DOSY spectra of pseudo-3D *D*-SMOOSY spectrum at two *s*patial *z*-points, the dimensions of gradient strengths were processed by exponential curve fitting using Supplementary Equation 2 on Dynamics Center 2.5 software.

The homogeneous sample was also applied to NMR experiments to investigate the effects of the different MAS frequencies to diffusion coefficient. The traditional pseudo-2D DOSY spectra of homogeneous sample were recorded at a MAS frequency of 3 kHz and 6 kHz at 299 K. The traditional DOSY experiment was performed by same NMR experimental parameters in previous paragraph.

Sample preparation for solution- and solid-state NMR experiments

For the solution-state NMR experiments, approximately 30 mg of a lyophilized shrimp sample was crushed to a powder using an Automill machine (Tokken, Inc., Chiba, Japan) at 1,400 rpm for 3 min. The crushed shrimp sample was suspended in 600 µL phosphate buffer solution (0.1 M K_2HPO_4/KH_2PO_4 (KPi); pH 7.0), containing 99% D_2O and 1 mM DSS-d6 as an internal standard for the NMR spectroscopy, and was incubated at 65°C for 15 min. The shrimp sample was then centrifuged (15,000 rpm; 4°C; 10 min), and the supernatant was collected in a 5-mm NMR tube. For membrane filtration experiments using *D*-SMOOSY, alanine, sucrose, and 140 mg polyvinyl alcohol (PVA; YAMATO Co., LTD., Tokyo, Japan) were suspended in 0.1 M KPi/D_2O buffer with 1 mM DSS-d6; alanine and sucrose were prepared to a final concentration of 12.5 mM in 1 mL buffer. Four sheets of polytetrafluoroethylene (PTFE) membrane filter (Merck KGaA, Darmstadt, Germany) with a 1 µm pore size were stacked and put into the 5 mm NMR tube so as to be about 2 cm from the bottom. After this, prepared sample was put on the membrane filter, and NMR measurements were started after 9 h. For feed diffusion, approximately 50 mg Himezakura fish feed pellets (HIGASHIMARU Co., Ltd., Kagoshima, Japan) were placed into 5-mm NMR tubes after freeze-drying. Thereafter, 500 µL 0.1 of M KPi/D_2O buffer with 1 mM DSS-d6 was added. *D*-SMOOSY measurements began 9 h after sample preparation.

Non-protease and protease processed shrimp samples were used for the solid-state NMR experiments. To prepare the non-protease processed sample, the lyophilized shrimp sample was crushed

to a powder and put into the MAS rotor (4-mm diameter). To prepare the protease processed shrimp sample, a powdered shrimp sample was prepared by using 2 mL of 0.1 M KPi containing 10 μL of protease (final concentration ≤ 0.25 μ/g) from *Aspergillus oryzae* (SIGMA). After reacting overnight at 37 °C and centrifugation at 15,000 rpm for 5 min, the residue was blown off with a SpeedVac vacuum concentrator (Thermo Fisher Scientific K.K., Tokyo, Japan) and dried. The shrimp sample was prepared by adding 2N HCL, leaving it at room temperature for 6 h and then washing with D_2O. Then 1N NaOH was added and the shrimp sample left for 6 h at 95°C before washing with D_2O. Finally, 95% ethanol was added and the shrimp sample was kept at room temperature for 6 h. The dried powder shrimp sample was put into MAS rotor.

Solution- and solid-state NMR experiments using shrimp

The solution-state NMR spectra of shrimp were recorded using an Avance II-700 spectrometer (Bruker, Billerica, MA) equipped with an inverse triple-resonance cryogenic probe with a *z*-axis gradient for 5-mm diameter samples; this was operated at 700.15 MHz for ^{1}H and 176.06 MHz for ^{13}C. ^{1}H–^{13}C heteronuclear single quantum coherence (HSQC), HSQC-total correlation spectroscopy (HSQC-TOCSY), and $REST_1$ spectra were recorded at 298 K for extraction from shrimp. The HSQC analysis was performed to confirm the existence of general compounds known to be present in shrimps. For this, 256 complex F1 (^{13}C) and 1024 complex F2 (^{1}H) points were recorded from 32 scans per F1 increment. The spectral widths obtained for F1 and F2 were 140 ppm and 14 ppm, respectively. The HSQC-TOCSY analysis was performed to evaluate the validity of signal annotation. For this, 256 complex F1 (^{13}C) and 2048 complex F2 (^{1}H) points were recorded from 64 scans per F1 increment. The spectral widths obtained for F1 and F2 were 140 ppm and 16 ppm, respectively. The mixing time using decoupling in the presence of scalar interactions 2 (DIPSI-2) as a spin lock pulse was 60 ms. The $REST_1$ experiment evaluated measurement performance and relaxation using modified DIPSI-2 to Malcolm Levitt 17 sequence (MLEV-17) as a spin lock pulse. The mixing time was 100 ms. The 180° selective refocusing pulse (REBURP) was used for the band selective pulse, and the methyl group with peaks in the range 1.0–0.6 ppm was excited. The 32 increments in the variable delay (VD) list used were 0.01, 0.02, 0.03, 0.04, 0.05, 0.06, 0.07, 0.08, 0.09, 0.1, 0.2, 0.3, 0.4, 0.5, 0.6, 0.7, 0.8, 0.9, 1.0, 1.5, 2.0, 2.5, 3.0, 3.5, 4.0, 4.5, 5.0, 5.5, 6.0, 6.5, 7.0, and 7.5 s. The recoded data points and spectral width on the F2 (^{1}H) axis were 16384 and 15 ppm from 96 scans.

The solid-state NMR spectra of shrimp were recorded using an Avance III HD-500 instrument (Bruker Corp., Billerica, MA) equipped with a double-resonance 4.0-mm MAS probe operating at 500.13 MHz for ^{1}H and 125.76 MHz for ^{13}C. Frequency switched Lee–Goldburg heteronuclear correlation (FSLG-HETCOR)[6], cross-polarization (CP)-T_1, and CP-$T_{1\rho}$ spectra were recorded by MAS at a frequency of 12 kHz at 299 K. The contact time was 200 μs for FSLG-HETCOR and 1 ms for CP-T_1, and CP-$T_{1\rho}$, with a 5-s recycling delay. The spectral width of the direct dimension in these

experiments was 299 ppm with 2048 points, and the spectral width of the indirect dimension for FSLG-HETCOR was 25 ppm with 64 points. For CP-T_1, the 16 increments in the VD list used were 0.001, 0.003, 0.005, 0.007, 0.01, 0.03, 0.05, 0.07, 0.1, 0.3, 0.5, 0.7, 1, 3, 5, and 7 s. For CP-$T_{1\rho}$, the 16 increments in the VP list used were 10, 25, 50, 75, 100, 250, 500, and 750 μs, and 1, 2.5, 5, 7.5, 10, 15, 20, and 30 ms. The Lee–Goldberg radio frequency (LG-RF) field was 71428 Hz, and small phase incremental alteration decoupling (71.4 kHz) was employed during acquisition. The CP-T_1 and CP-$T_{1\rho}$ spectra obtained by solid-state NMR experiment and the $REST_1$ spectra obtained by solution-state NMR were processed using Dynamics Center 2.5 software (https://www.bruker.com/).

Solution-state NMR experiments for heterogeneous systems

The solution-state NMR spectra of heterogeneous systems were recorded using an Avance II-700 spectrometer (Bruker, Billerica, MA) equipped with an inverse triple-resonance cryogenic probe with a *z*-axis gradient for 5-mm diameter samples; this was operated at 700.15 MHz for ^{1}H and 176.06 MHz for ^{13}C. *D*-SMOOSY spectra were recorded at 298 K for fish feed sample and PTFE membrane sample as other application examples. The *D*-SMOOSY analysis was performed to evaluate fish feed diffusion and PTFE membrane filtration. For this, 32 complex F1 (^{1}H) points, 2048 complex F3 (^{1}H) points, and 14 points of gradient strength on F2 were recorded from 16 scans per F1 and F2 increment. The spectral widths obtained for F3 was 12 ppm. The maximum gradient strength for F1 was 2.41 G/cm, and the minimum gradient strength for F1 was −2.41 G/cm; the 32 increments had equal intervals. The 14 increments in the diffusion list used for F2 were 0.963, 4.408, 7.852, 11.297, 14.741, 18.186, 21.63, 25.075, 28.52, 31.964, 35.409, 38.853, 42.298, and 45.743 G/cm. The diffusion time was 60 ms.

The experimental parameters for CSI and SMOOSY using intact shrimp

The maximum gradient strength for spatial *z*-profiling was 9.63 G/cm for CSI, *D*-SMOOSY, T_1-SMOOSY, and T_2-SMOOSY, and 4.815 G/cm for REST-SMOOSY. The increment in gradient strength for spatial *z*-profiling in SMOOSY was 0.15 G/cm. For *D*-SMOOSY, the 24 increments in the *difflist* file used were 0.963, 2.910, 4.857, 6.804, 8.751, 10.698, 12.645, 14.592, 16.538, 18.485, 20.432, 22.379, 24.326, 26.273, 28.220, 30.167, 32.114, 34.061, 36.008, 37.955, 39.902, 41.849, 43.796, and 45.742 G/cm. For T_1-SMOOSY, the 12 increments in the VD list used were 0.01, 0.02, 0.04, 0.08, 0.1, 0.2, 0.4, 0.8, 1, 2, 4, and 8 s. For $REST_1$-SMOOSY, the eight increments in the VD list used were 0.04, 0.06, 0.2, 0.4, 0.6, 2.0, 4.0, and 6.0 s. For T_2-SMOOSY and $REST_2$-SMOOSY, the 12 increments in the variable counter (VC) list were 12, 24, 36, 48, 60, 72, 84, 96, 108, 120, 132, and 144 loop counters (Supplementary Table 3).

^{1}H–^{13}C heteronuclear single quantum coherence (HSQC) spectroscopy was also employed for signal annotation when using the SpinAssign tool in InterSpin[7] (http://dmar.riken.jp/interspin/). For this, 96 complex F1 (^{13}C) and 1024 complex F2 (^{1}H) points were recorded from 8192 scans per F1

increment. Moreover, 50% of data point on indirect dimension (F2) was collected by non-uniform sampling technique. The spectral widths obtained for F1 and F2 were 80 ppm and 16 ppm, respectively. The HSQC spectrum was recorded by MAS at a frequency of 3 kHz at 299 K. The indirect dimension was processed by the compressed sensing method in Bruker's Topspin software (https://www.bruker.com/).

PARAFAC of pseudo-3D SMOOSY spectra of intact shrimp

The three-way parallel factor analysis (PARAFAC) facilitates extraction of the features from 3D data, for comparison the results with principal component analysis (PCA) using 2D data, PARAFAC using pseudo-3D SMOOSY spectra was performed by parafac function in multiway package on R software.

B. Supplementary Discussion

Evaluation of biopolymers in shrimp using solid-state NMR

CSI and SMOOSY using HR-MAS did not detect the chitin that constitutes the shell of the shrimp. This may have been due to the very short T_2. A recent study of relaxation by solid-state NMR using the chitin of intact *A. fumigatus* cell walls reported that ^{1}H-$T_{1\rho}$ was 4.5 ms[8]. In the present study, therefore, the polymers containing chitin were evaluated by multidimensional CP experiments using solid-state NMR. Assigning chitin was performed using the FSLG-HETCOR spectrum with reference from previous studies[9,10]. The assignment of chitin signals was possible with the protease-treated shrimp sample (Supplementary Figure 8a); these were predominantly of β-chitin, with α-chitin hardly detectable. With the shrimp sample that was not treated with protease, the protein signals were predominant, and some β-chitin signals were also confirmed (Supplementary Figure 8b). β-Chitin signals were also confirmed with CP-T_1 using the protease-treated shrimp sample; the ^{1}H-T_1 time of all the signals was the same time, at 2.05 s (Supplementary Figure 8c). These results confirmed the presence of β-chitin in shrimps. The signals for β-chitin were also confirmed with CP-$T_{1\rho}$ using a non-protease-treated shrimp sample; ^{1}H-$T_{1\rho}$ was approximately 4 ms, similar to that previously reported[8] (Supplementary Figure 8d). The different relaxation times at each site suggested that there may have been an effect due to the interaction with protein. Thus, in solid-state NMR experiments, it was possible to evaluate chitin as a biopolymer that could not be obtained in SMOOSY experiments using HR-MAS. Moreover, in solid-state NMR, 3D imaging method using magic sandwich echo has been proposed[11]. By applying this method to SMOOSY, we could expect to observe the spatial physical properties of biopolymers. However, there are few reported cases and it can be said that this is a developing technology. In the future, it is expected that the application of these techniques will make it possible to evaluate the compounds and their diffusion or relaxation of polymers in intact biological samples.

Evaluation of compounds in shrimp using solution-state NMR

Solution-state NMR was used to measure compounds generally observed in shrimp. The signal annotation of the complex mixture was performed using ^{1}H–^{13}C HSQC spectra and chemical shift databases, along with the results from previous studies[10,12]. This confirmed the presence of 30 compounds, including amino acids, organic acids, and saccharides (Supplementary Figure 10a). Trimethylamine N-oxide (TMAO) and betaine are substances that adjust the osmotic pressure[13] and are unique NMR signals in crustaceans. Taurine has been observed in the free state in various animal and plant tissues[14]. The spin lock pulse of the original REST was changed from DIPSI-2 to MLEV-17, and this modified pulse sequence was used to evaluate the measurement performance. The methyl groups of three amino acids (leucine, isoleucine, and valine) was excited by the REBURP shape and measured. Signal separation is possible in the T_1 axis of the indirect dimension even when the compounds have

similar chemical structures (Supplementary Figure 10b). The experimental values of T_1 for the methyl groups of leucine, isoleucine, and valine were 0.87, 1.02, and 1.15 s, respectively. The rotational correlation time was obtained from a molecular dynamics calculation using Gromacs 5.0.4 software, and a theoretical value of the vertical relaxation time was calculated based on the Bloch–Redfield–Wangsness theory[15]. The experimental and theoretical relaxation times were confirmed to be similar.

C. Supplementary Figures and Tables

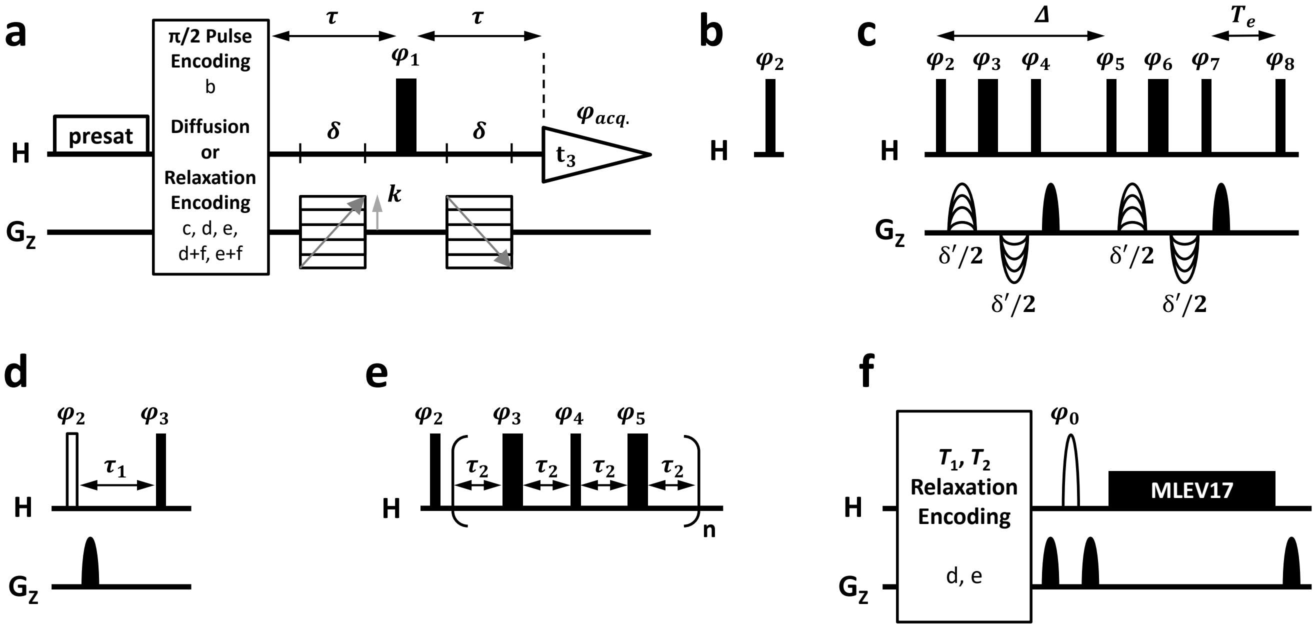

Supplementary Figure 1 Pulse sequences. **a+b** CSI, SMOOSY driven by (**a, c)** diffusion encoding (*D*-SMOOSY, as well as CSI-DOSY), (**a, d)** T_1 encoding (T_1-SMOOSY, as well as CSI-SR), (**a, e)** T_2 encoding (T_2-SMOOSY, as well as CSI-PROJECT), (**a, d, f)** $REST_1$ encoding ($REST_1$-SMOOSY, as well as CSI-$REST_1$), and (**a, e, f)** $REST_2$ encoding ($REST_2$-SMOOSY, as well as CSI-$REST_2$). The presat employs continuous wave (CW) and randomization pulse. The CW method is used for solvent suppression, and a randomization pulse is used to dephase residual magnetization[16]. The black narrow and wide rectangles denote hard 90° and 180° RF pulses and produce a spin echo at time τ. **a** A magnetic field gradient pulse of length δ and strength k encodes the signal with respect to position. **c** Big delta Δ is the diffusion time and the length of little delta δ' is diffusion gradient; gradient levels are changed to characterize diffusion or relaxation. *Te* indicates eddy current delay. **d** The white rectangle pulse with dephasing gradient pulse is used for saturation, and τ_1 denotes T_1 recovery time. **e** The echo time (τ_2) is delay for relay of echo. **f** The white shape denotes band selective pulse with MLEV-17 as the spin lock pulse used for TOCSY experiment. SR, saturation recovery; PROJECT, periodic refocusing of *J* evolution by coherence transfer. These pulse programs are available to obtain from Supplementary Data 1-6.

Supplementary Table 1 Phase cycling for the CSI and SMOOSY sequences shown in Supplementary Figure 1.

	2D CSI<br>**a+b**	Pseudo-3D<br>*D*-SMOOSY<br>**a+c**	Pseudo-3D<br>T_1-SMOOSY<br>**a+d**	Pseudo-3D<br>T_2-SMOOSY<br>**a+e**	Pseudo-3D<br>$REST_1$-SMOOSY<br>**a+d+f**	Pseudo-3D<br>$REST_2$-SMOOSY<br>**a+e+f**
φ_0	-	-	-	-	x, y, -x, -y	x, y, -x, -y
φ_1	x, -x, $(y, -y)_2$, -x, x, y, -y, $(-x, x)_2$, -y, y	$(x, -x)_2$, $(-x, x)_2$, $(y, -y)_2$, $(-y, y)_2$	x	x	x	x
φ_2	$x_4, y_4, -x_4, -y_4$	x	x, -x	x, -x	$x_4, -x_4$	$x_4, -x_4$
φ_3	-	x	$x_2, -x_2$	$y_2, -y_2$	$x_8, -x_8$	y
φ_4	-	$x_2, -x_2$	-	$y_4, -y_4$	-	y
φ_5	-	$x_4, -x_4, y_4, -y_4$	-	$y_2, -y_2$	-	y
φ_6	-	x	-	-	-	-
φ_7	-	$(x, -x)_2$, $(-x, x)_2$, $(y, -y)_2$, $(-y, y)_2$	-	-	-	-
φ_8	-	$x_4, -x_4, y_4, -y_4$	-	-	-	-
$\varphi_{acq.}$	$x_2, -x_2, y_2, -y_2$	x, $-x_2$, x, -x, x_2, -x, -y, y_2, -y, y, $-y_2$, y	$x_2, -x_2$	$(x, -x)_2$	$(x, -x)_4$, $(-x, x)_4$	$(x, -x)_2$, $(-x, x)_2$

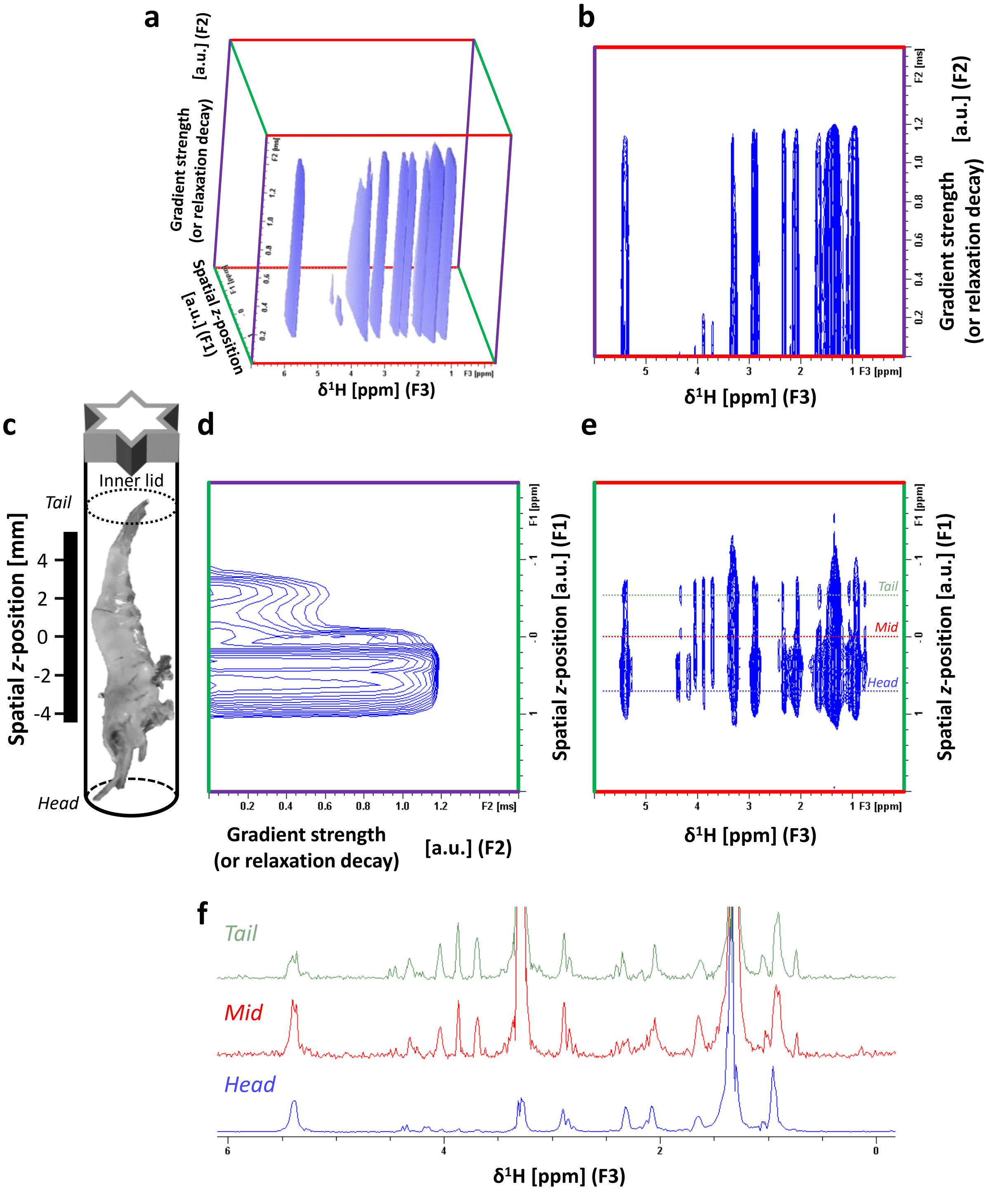

Supplementary Figure 2 An example of a pseudo-3D SMOOSY spectrum. **a** pseudo-3D *D*-SMOOSY spectrum for an intact shrimp created using Topspin software. The F3 and F1 axes were Fourier transformed. The red axis (F3) represents the ^{1}H chemical shift, and the purple axis (F2) represents the gradient strength. For T_1-SMOOSY, T_2-SMOOSY, $REST_1$-SMOOSY, and $REST_2$-SMOOSY, the F2 axis shows the relaxation decay and the green axis (F1) shows the spatial *z*-profile. The 2D projections of the pseudo-3D spectrum are shown in **b**, **d**, and **e**. **b** The projected F3 vs. F2 spectrum shows the mean results for 2D DOSY. **c** A shrimp and deuterated methanol buffer in the HR-MAS rotor. The black bar shows the range of detected spatial *z*-position. **d** The projected F2 vs. F1 spectrum shows the mean results for 2D diffusion-weighted imaging. **e** The projected F3 vs. F1 spectrum shows the mean results for 2D CSI. **f** The sliced 1D ^{1}H spectra of body components on CSI spectrum **e** shows spectral quality. This experiment was conducted using HR-MAS probe at a MAS frequency of 3 kHz at 299 K. The details of experimental parameters are shown in Supplementary Tables 3 and 4. This spectrum was processed and drawed on TopSpin software, and the spatial *z*-position was not corrected. This spectrum was spectrum before process by SMOOSY processor. The spatial *z*-position could be corrected by SMOOSY processor, and shown in Figure 2 and Supplementary Figure 9c.

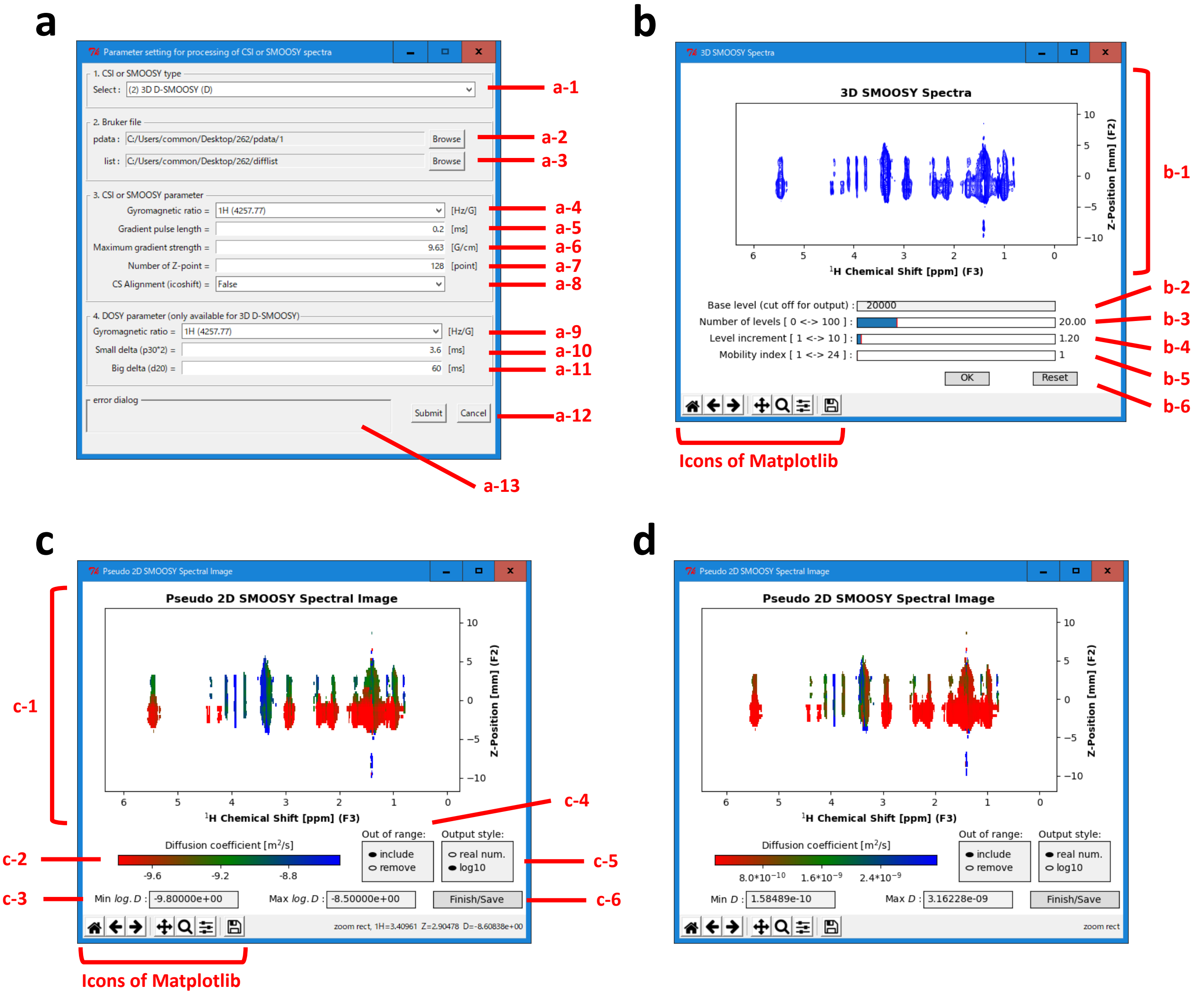

Supplementary Figure 3 Details of SMOOSY processor developed for pseudo-3D SMOOSY to pseudo-2D SMOOSY spectral image. **a** The first main window shows the parameter settings for processing the spectra. **a-1** 2D CSI and pseudo-3D SMOOSY (*D*-SMOOSY, T_1-SMOOSY, T_2-SMOOSY, REST$_1$-SMOOSY, and REST$_2$-SMOOSY) can be chosen as the process. **a-2**, **a-3** The Bruker pdata file and list file (difflist: for *D*-SMOOSY, vdlist: for T_1-SMOOSY, T_2-SMOOSY, REST$_1$-SMOOSY, and REST$_2$-SMOOSY) are required. The axes for the chemical shift and spatial *z*-profile should be Fourier transformed, but the diffusion or relaxation dimension (diffusion or relaxation) should not be processed when this input. **a-4** ^{1}H and ^{13}C are available to select as the gyromagnetic ratio for observed nucleus. **a-5**, **a-6** The gradient pulse length (δ of CSI in Supplementary Figure 1a) and the maximum gradient strength (*k* of CSI in Supplementary Figure 1a) depend on the experiment and instrument. **a-7** The size of fid for the spatial *z*-profile is set according to the experiment. **a-8** Chemical shift alignment using icoshift is also available. **b** The plot window for the selected pseudo-3D SMOOSY with the selected parameter setting. **b-1** The spectra of chemical shift versus spatial *z*-position are plotted in the upper part of the screen. The contour plot parameters (base level, number of levels, level increment, and diffusion or relaxation index of the third dimension) are set by scores (from **b-2** to **b-5**). **b-2** Lower limit value of signal intensity is displayed in contour line. The noise signals can be cut off in the section. **b-6** Processing of the diffusion or relaxation dimension starts when the "OK" button is clicked; the "Reset" button returns to the initial state. Matplotlib icons provide the plot functions for zooming, saving the figure, etc. **c** The plot window showing the processed SMOOSY spectra. **c-1** The spectral image of chemical shift versus spatial *z*-position is plotted in the upper part of the screen according to the. **c-2** The signals are colored by diffusion coefficient for *D*-SMOOSY or the relaxation time for T_1-SMOOSY, T_2-SMOOSY, REST$_1$-SMOOSY, and REST$_2$-SMOOSY. The range of diffusion or relaxation is controlled by section **c-3**. How signals that are out of diffusion or relaxation range are plotted can be selected in section **c-4**. **c-5** Diffusion coefficients for *D*-SMOOSY and relaxation time for T_1-SMOOSY, T_2-SMOOSY, REST$_1$-SMOOSY, and REST$_2$-SMOOSY can be scaled by logarithm. **d** SMOOSY can be also scaled by integer, but may not show significant spatial differences compared to logarithm scale. **c-6** The 2D matrix data of the processed spectra can be exported to the "pdata/No." directory by clicking the "Finish/Save" button. The executable file or original python code of SMOOSY processor is available from http://dmar.riken.jp/Rscripts/.

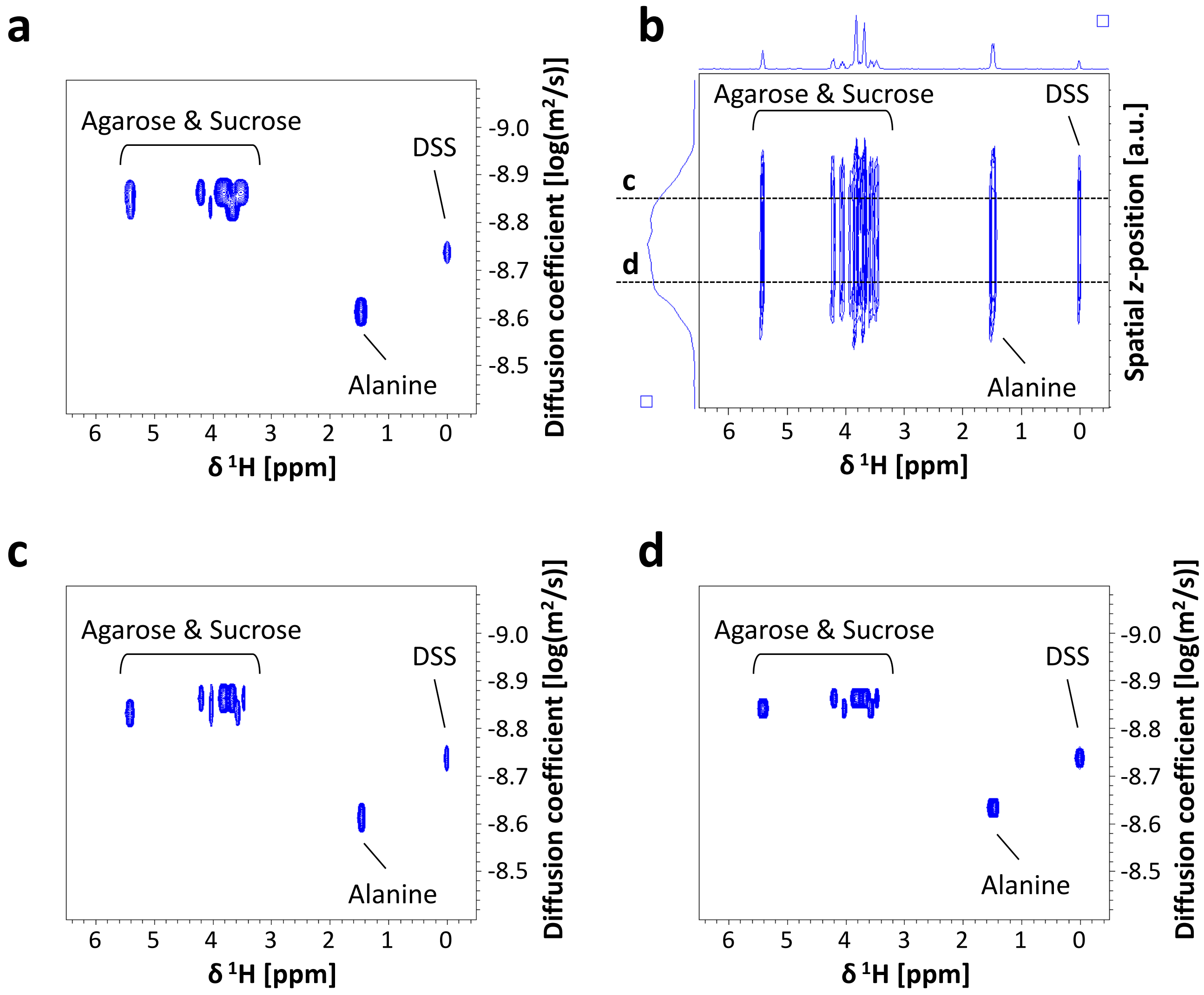

Supplementary Figure 4 A comparison between traditional pseudo-2D DOSY spectrum and sliced pseudo-2D DOSY spectra from pseudo-3D *D*-SMOOSY spectrum of homogeneous sample. These spectra were collected by 3 kHz MAS frequency at 299K. The details of experimental parameters are shown in Supplementary Tables 3 and 4. The dimension of gradient strength was processed by mono-exponential curve fitting[3]. **a** Traditional pseudo-2D DOSY spectrum. **b** Projected CSI spectrum of 128 planes in pseudo-3D *D*-SMOOSY spectrum. This spectrum was processed and drawn on TopSpin software, and the spatial *z*-position was not corrected. This spectrum was spectrum before process by SMOOSY processor. **c** Pseudo-2D DOSY spectrum of sliced upper-side plane of **b**, and **d** pseudo-2D DOSY spectrum of sliced lower-side plane of **b**.

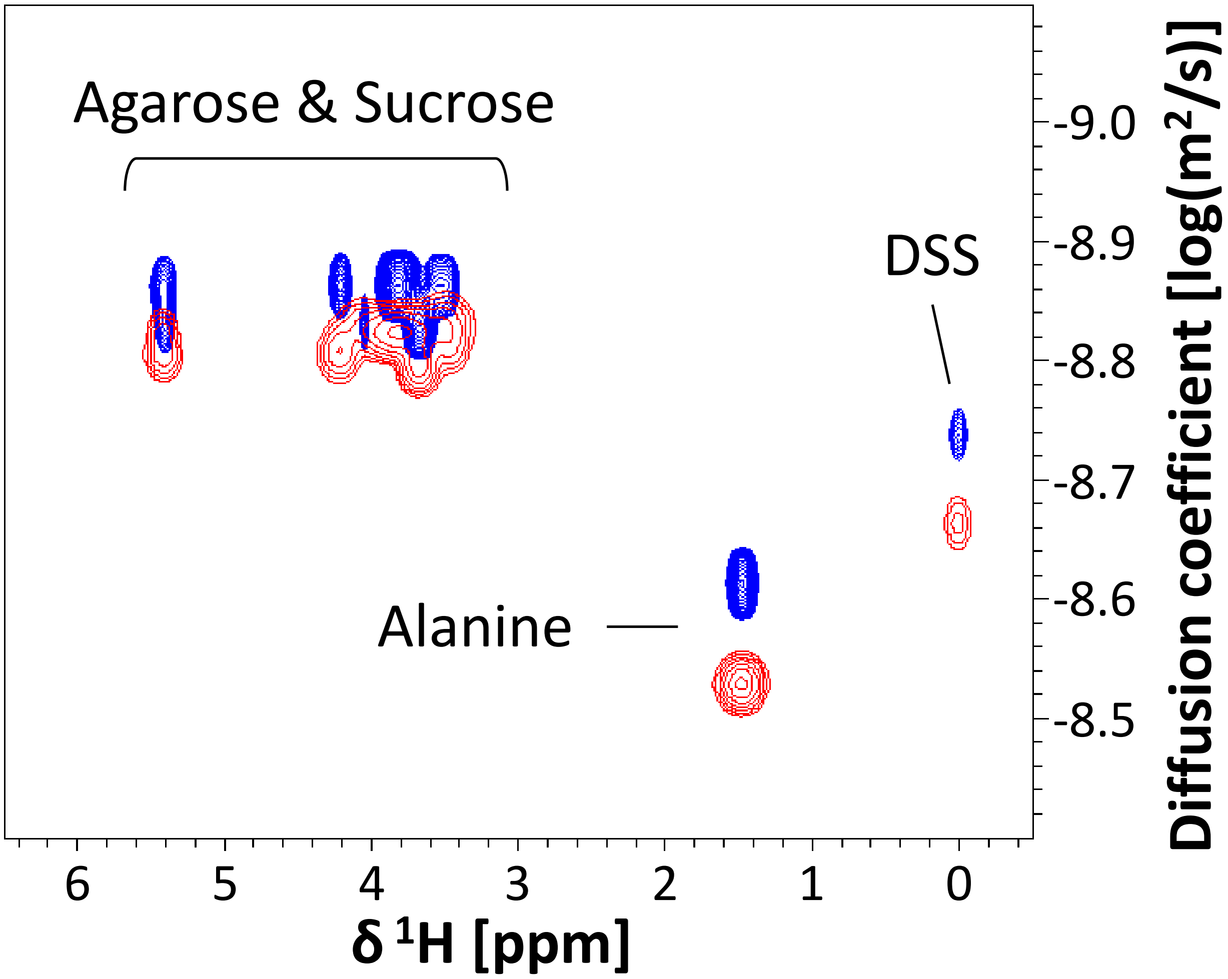

Supplementary Figure 5 Pseudo-2D DOSY spectra of homogeneous samples with different MAS frequency. Blue signals were recorded by 3 kHz MAS frequency at 299K, and the red signals were recorded by 6 kHz MAS frequency at 299K. The dimensions of gradient strength were processed by mono-exponential curve fitting. These spectra were processed by Dynamics Center and drawed on TopSpin software

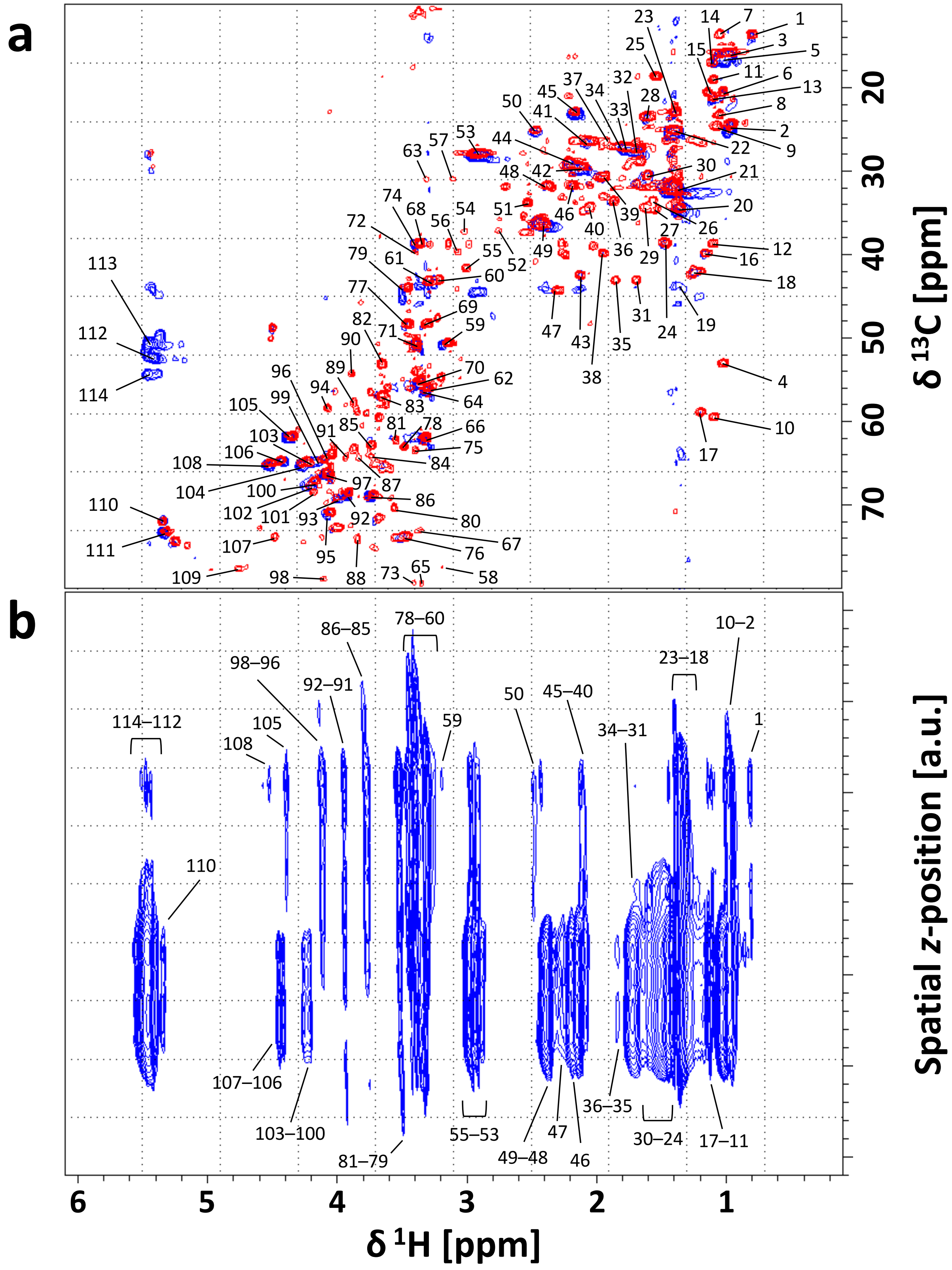

Supplementary Figure 6 a ^{1}H–^{13}C HSQC spectrum of an intact shrimp (blue) and ^{1}H–^{13}C HSQC spectrum of extract from powdered shrimp (red) with a deuterated methanol (MeOD) buffer. **b** CSI spectrum of an intact shrimp with a MeOD buffer. HSQC and CSI experiments were measured using HR-MAS probe at a MAS frequency of 3 kHz at 299 K. The peak numbers represent the annotated chemical shifts by the SpinAssign tool in InterSpin[7]. (listed in Supplementary Table 2). This spectrum was processed and drawed on TopSpin software, and the spatial *z*-position was not corrected. This spectrum was spectrum before process by SMOOSY processor. The spatial *z*-position could be corrected by SMOOSY processor, and shown in Supplementary Figure 9b.

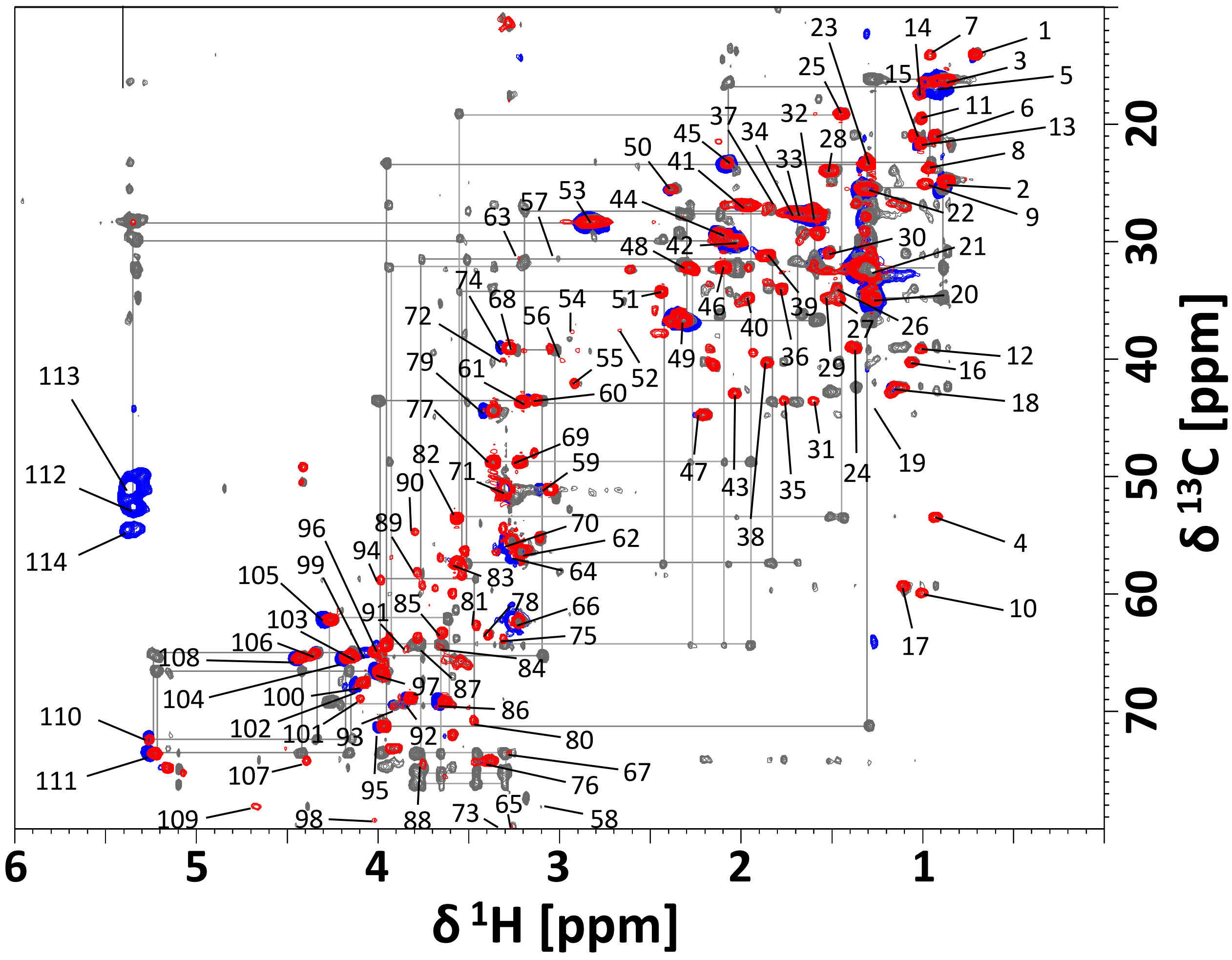

Supplementary Figure 7 ^{1}H–^{13}C HSQC spectrum of an intact shrimp (blue), ^{1}H–^{13}C HSQC spectrum (red), and ^{1}H–^{13}C HSQC-TOCSY spectrum of extract from powdered shrimp (gray) with a MeOD buffer. HSQC was measured using HR-MAS probe at a MAS frequency of 3 kHz at 299 K. The peak numbers represent the annotated chemical shifts by the SpinAssign tool in InterSpin[7]. (listed in Supplementary Table 2). The gray lines show the chemical fragment network of same spin system by chain assignment using correlation signals of HSQC-TOCSY. The fragment network of signals from cholesterol is not shown because of complex network.

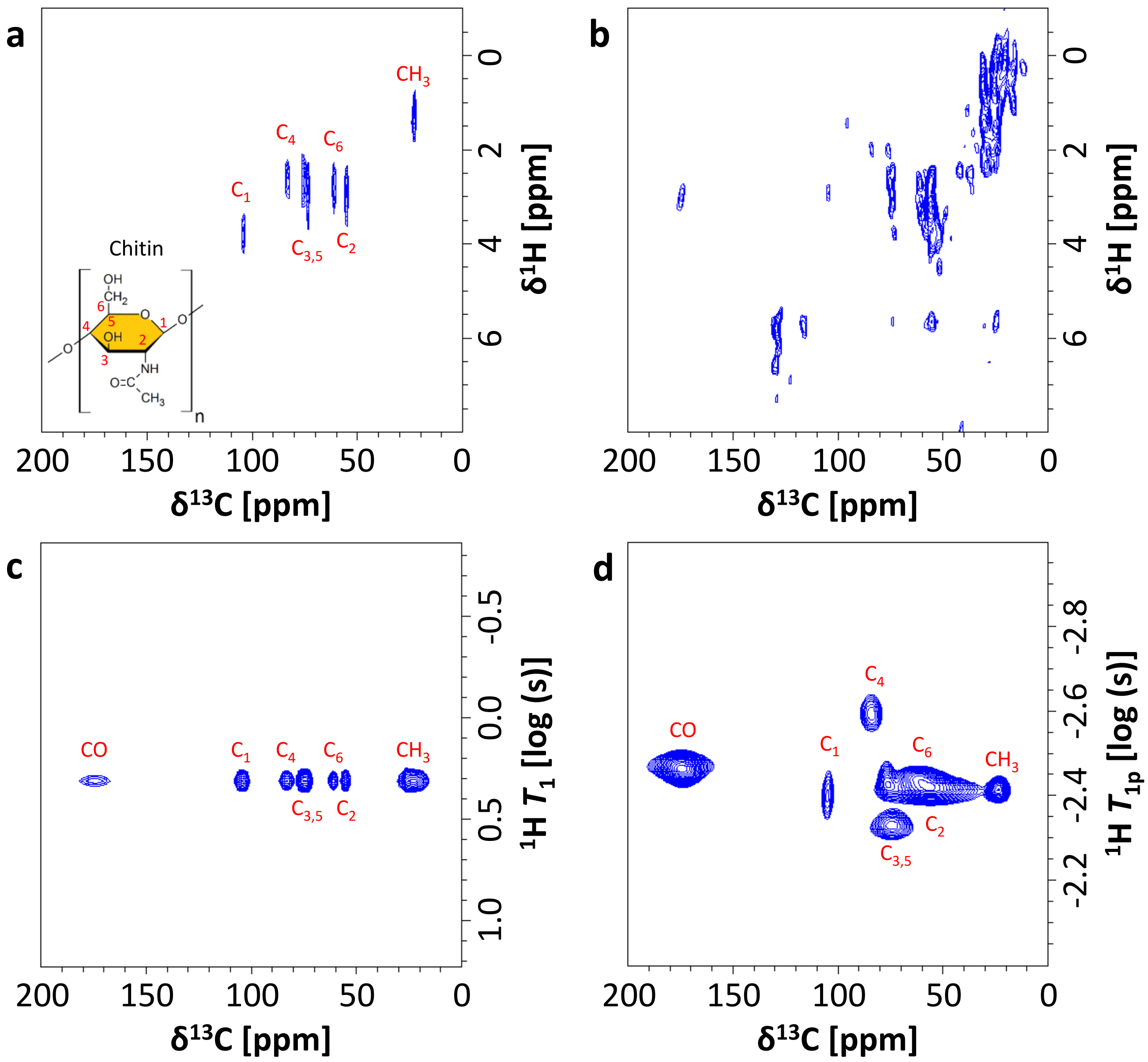

Supplementary Figure 8 Chitin, a major polymer bound in shrimp shells, could not be detected by solution-state NMR or HR-MAS, but was evaluated by solid-state NMR. FSLG-HETCOR was applied to powdered shrimp user **a** with or **b** without protease processing for the assignment of chitin. Polymer relaxation was evaluated **c** by CP-T_1 using a sample processed with protease and **d** by CP-$T_{1}\rho$ using a sample not processed with protease. These experiments were conducted using MAS at a frequency of 12 kHz at 299 K. The signals of chitin were assigned (δ^{1}H [ppm], δ^{13}C [ppm]; 22.68, 1.33 (CH_3); 55.09, 2.90 (C_2); 61.22, 2.81 (C_6); 73.48, 2.81 (C_3); 75.81, 2.71 (C_5); 83.11, 2.61 (C_4); 104.13, 3.79 (C_1); 174.26, - (CO)) from references[8,9]. The ^{1}H-T_1 was around 2.05 s and the ^{1}H-$T_{1}\rho$ was around 4 ms.

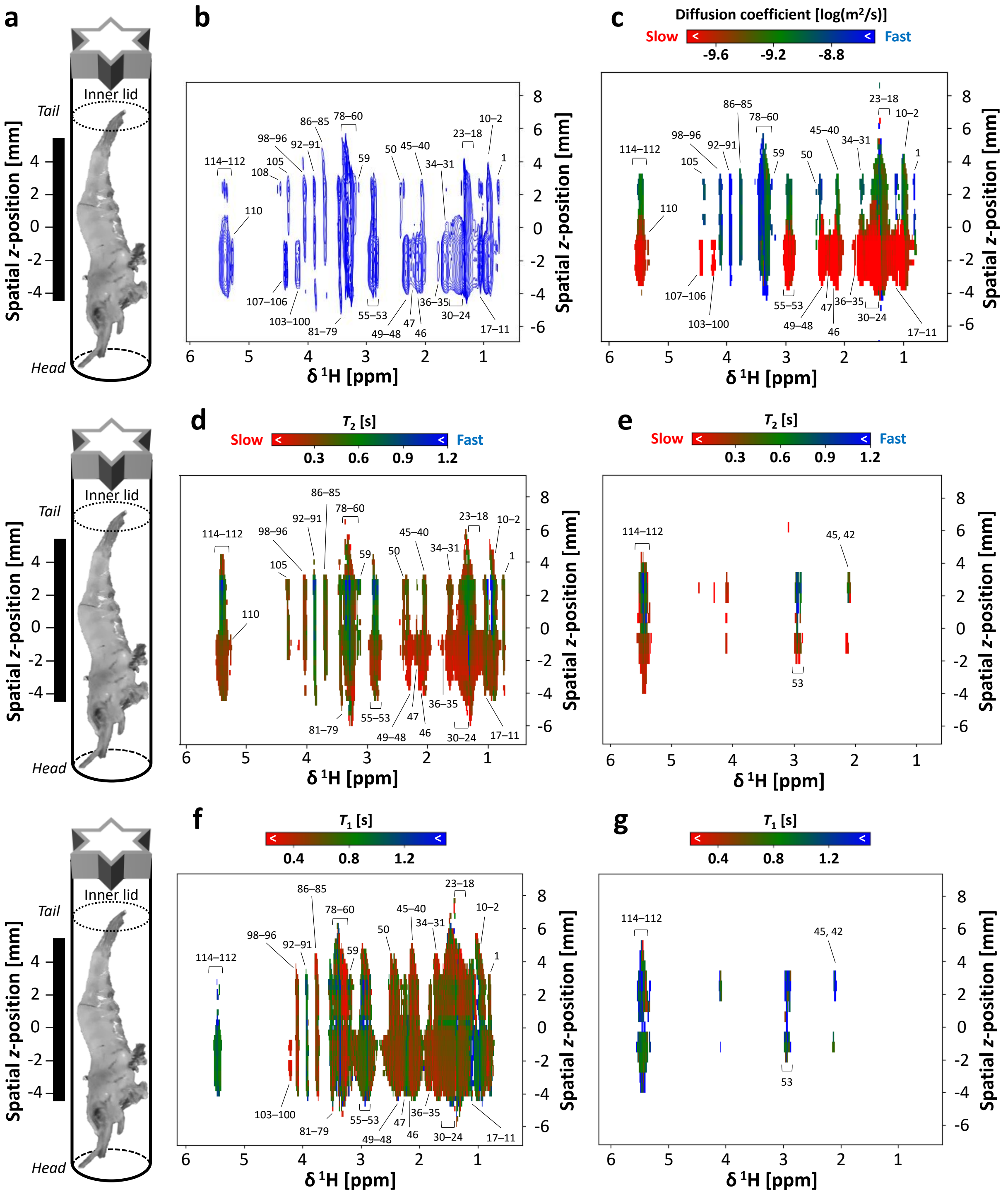

Supplementary Figure 9 2D CSI spectrum and pseudo-2D SMOOSY spectral image of an intact shrimp in the HR-MAS rotor. **a** The shrimp and MeOD buffer were put into the HR-MAS rotor. The black bars show the range of detected spatial *z*-position. **b** 2D CSI spectrum. The spatial *z*-position was calculated with SMOOSY processor. The compounds shown in **b** were annotated from the results of the annotated HSQC signals (see Supplementary Figure 6 and Supplementary Table 2). **c** The pseudo-2D *D*-SMOOSY spectral image, **d** the pseudo-2D T_2-SMOOSY spectral image, **e** the pseudo-2D REST$_2$-SMOOSY spectral image, **f** the pseudo-2D T_1-SMOOSY spectral image, and **g** the pseudo-2D REST$_1$-SMOOSY spectral image were processed from the pseudo-3D SMOOSY spectrum with SMOOSY processor. The band selective region by REBURP pulse for the REST experiment was 6–4.5 ppm; this targeted the olefin in fatty acids. The resolution of spatial *z*-position was 300 μm for **b**–**d** and **f**, and 600 μm for **e** and **g**. The color bars in **c**–**g** show the range of diffusion coefficients or relaxation times. The details of experimental parameters are shown in Supplementary Tables 3 and 4. These experiments were conducted using HR-MAS probe at a MAS frequency of 3 kHz at 299 K. T_2- and T_1-weighted parameters were repetition time (TR) = 2000 ms, echo time (TE) = 1 ms, number of excitations (NEX) = 24, and matrix 2048 × 128 (chemical shift × *z*-position). REST$_2$ and REST$_1$-weighted parameters were TR = 2000 ms, TE = 1 ms, NEX = 64, and matrix 2048 × 64 (chemical shift × *z*-position).

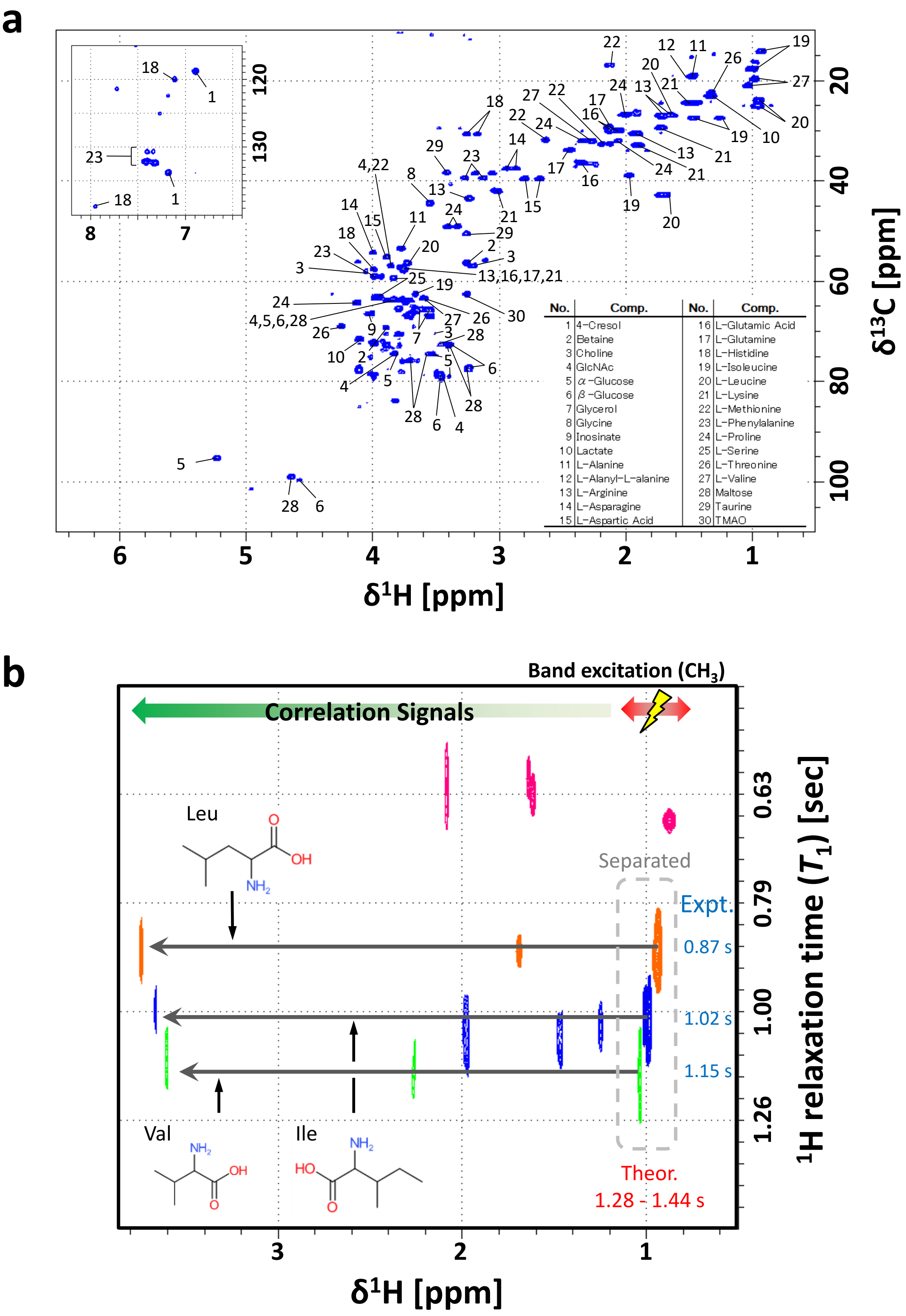

Supplementary Figure 10 The compounds of a shrimp in D_2O were evaluated by solution-state NMR. **a** ^{1}H–^{13}C HSQC spectroscopy was used to confirm the existence of general compounds known to be present in shrimps. The signals were annotated according to reference[10,12] and using the SpinAssign tool in InterSpin[7]. The upper left shows the expanded spectrum on the low magnetic field region. These annotated compounds are listed in the table at the bottom right of the spectrum. **b** $REST_1$[17], with DIPSI-2 modified to MLEV-17 as the spin lock pulse, was used to evaluate measurement performance and relaxation. A theoretical relaxation time was computed by molecular dynamics simulation based on the Bloch–Redfield–Wangsness theory[15]. The experimental and theoretical relaxation times were shown to be similar. The purple signals may be from proteins.

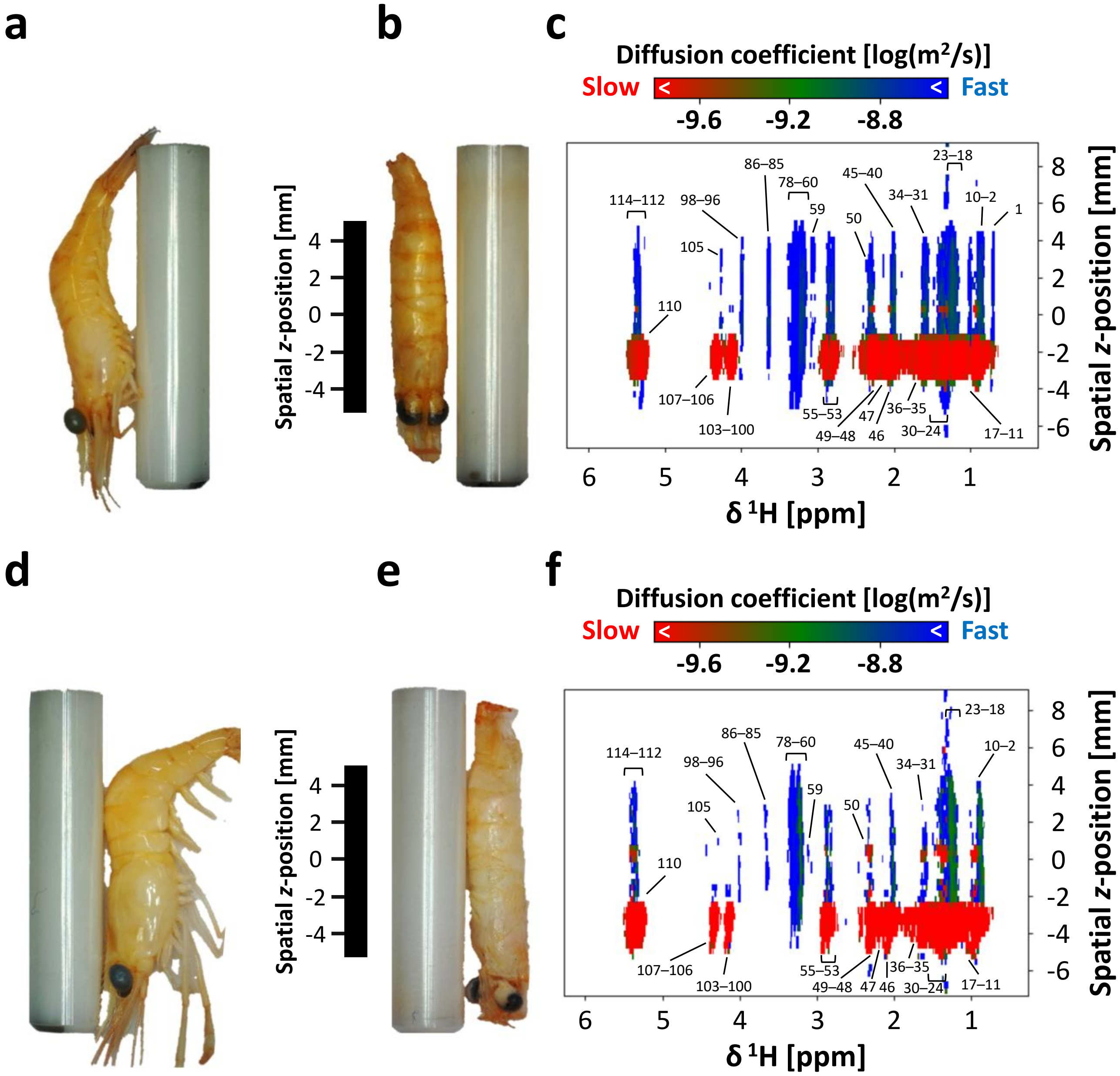

Supplementary Figure 11 *D*-SMOOSY experiments at different MAS frequency. Intact shrimp and HR-MAS rotor **a** before, and **b** after NMR experiments at 2 kHz MAS frequency at 299 K. **c** The pseudo-2D *D*-SMOOSY spectral image of **b**. Intact shrimp and HR-MAS rotor **d** before, and **e** after NMR experiments at 6 kHz MAS frequency at 299 K. **f** The pseudo-2D *D*-SMOOSY spectral image of **e**. The black bars show the range of spatial *z*-position. These experiments were conducted with same sample preparation and same parameters of NMR measurement shown in Supplementary Tables 3 and 4 **e**xcept for the MAS frequency. The antennas, legs, and a part of tail of shrimp were cut off before these experiments. The intact shrimp used in this experiment are two different individuals. The picture of intact shrimp was taken after approximately 48 h from MAS start.

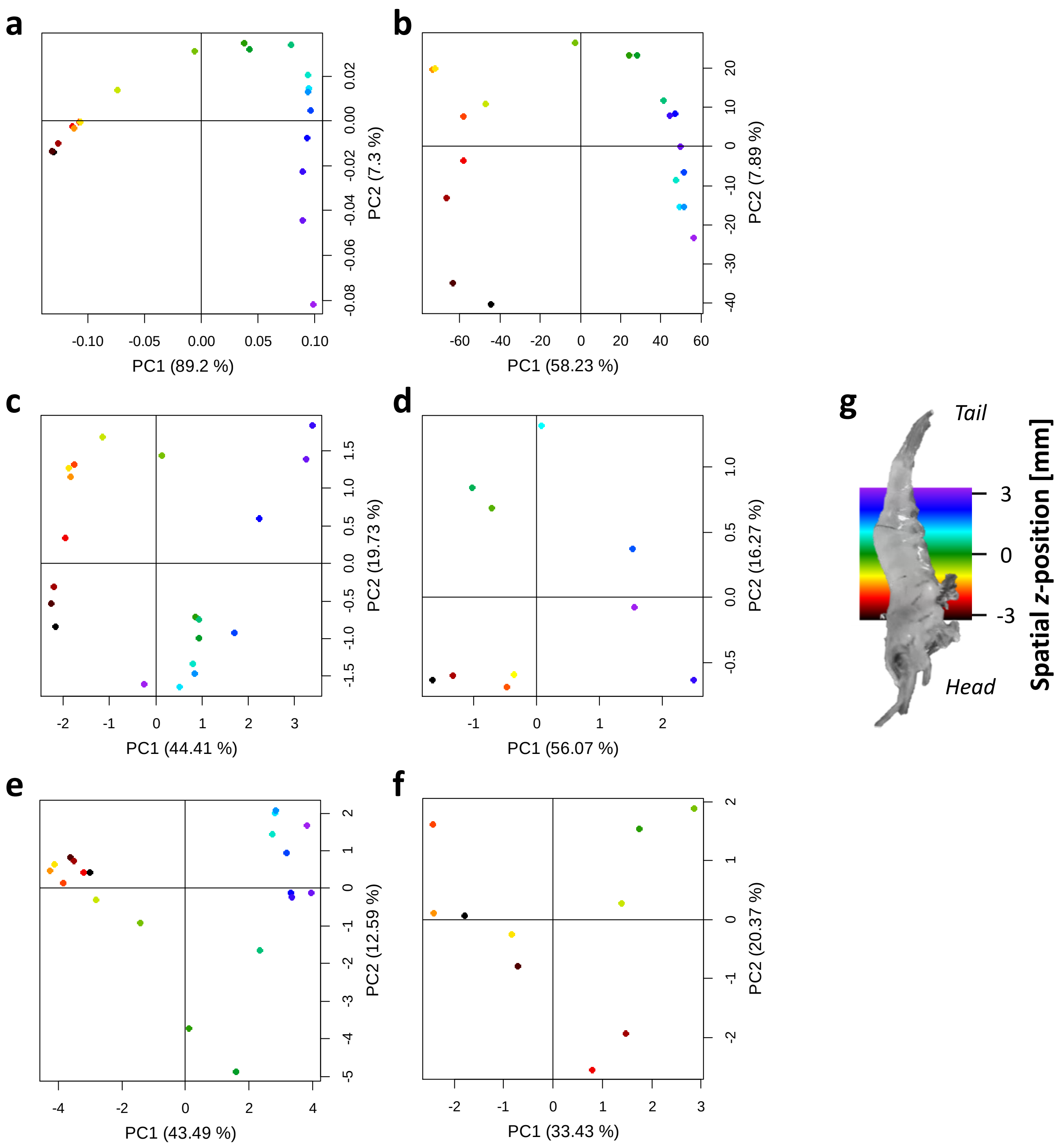

Supplementary Figure 12 Score plots for PC1 and PC2 from PCA using 2D CSI spectrum and pseudo-2D SMOOSY spectral image to extract the features of the spatial *z*-position. The distribution of intensity of CSI signals on the spatial *z*-position axis was normalized using the CSI spectra of a uniform standard sample for PCA. The PCA scores using **a** CSI, **b** *D*-SMOOSY, **c** T_2-SMOOSY, **d** $\mathrm{REST_2}$-SMOOSY, **e** T_1-SMOOSY, and **f** $\mathrm{REST_1}$-SMOOSY are colored according to the body part they represent, as shown in **g**.

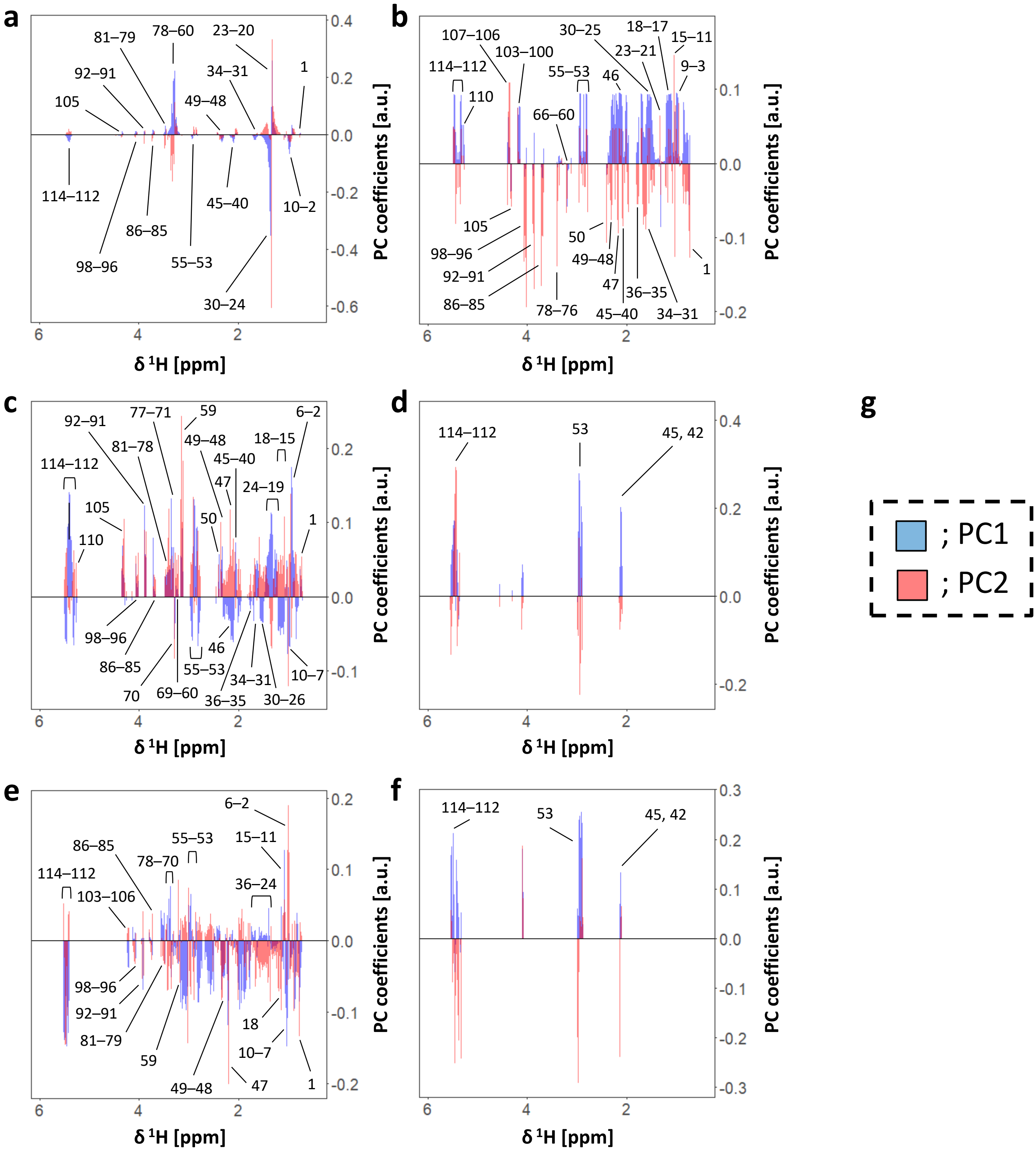

Supplementary Figure 13 Loading plots for PC1 and PC2 from PCA using 2D CSI spectrum and pseudo-2D SMOOSY spectral image. The loadings from PCA using **a** CSI, **b** *D*-SMOOSY, **c** T_2-SMOOSY, **d** REST_2-SMOOSY, **e** T_1-SMOOSY, and **f** REST_1-SMOOSY are colored **g** in blue for PC1 and red for PC2. The loadings were annotated according to the CSI signals in Supplementary Figure 6 and Supplementary Table 2.

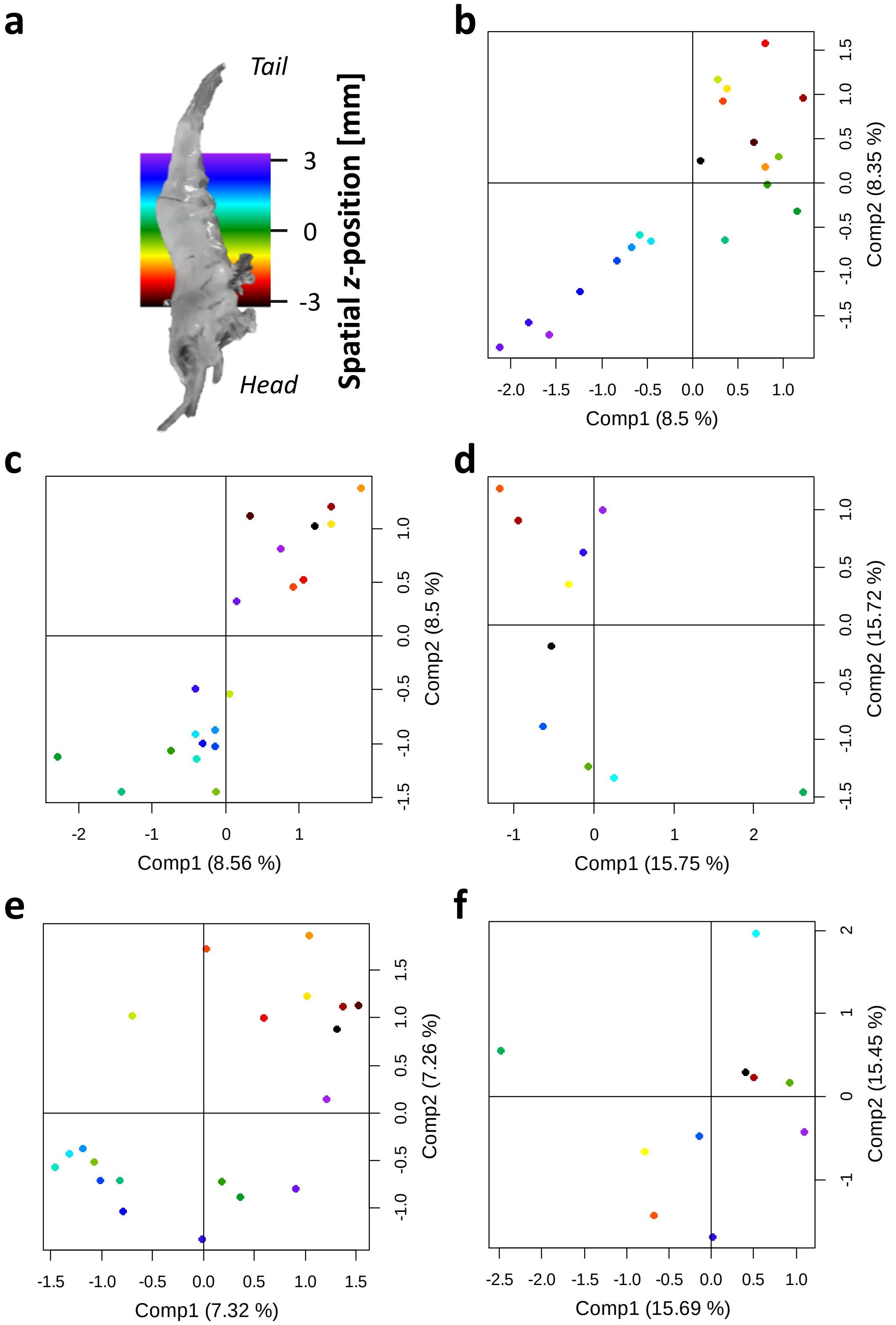

Supplementary Figure 14 Weight plots for Comp.1 and Comp.2 from the 3-way PARAFAC model using pseudo-3D SMOOSY spectra, the first mode scores (loadings in mode 1) in the matrix A contain feature at each spatial *z*-position. **a** The weights are colored according to the body part they represent. The weights were calculated using **b** pseudo-3D *D*-SMOOSY, **c** T_2-SMOOSY, **d** $REST_2$-SMOOSY, **e** T_{1}-SMOOSY, and **f** $REST_1$-SMOOSY, respectively.

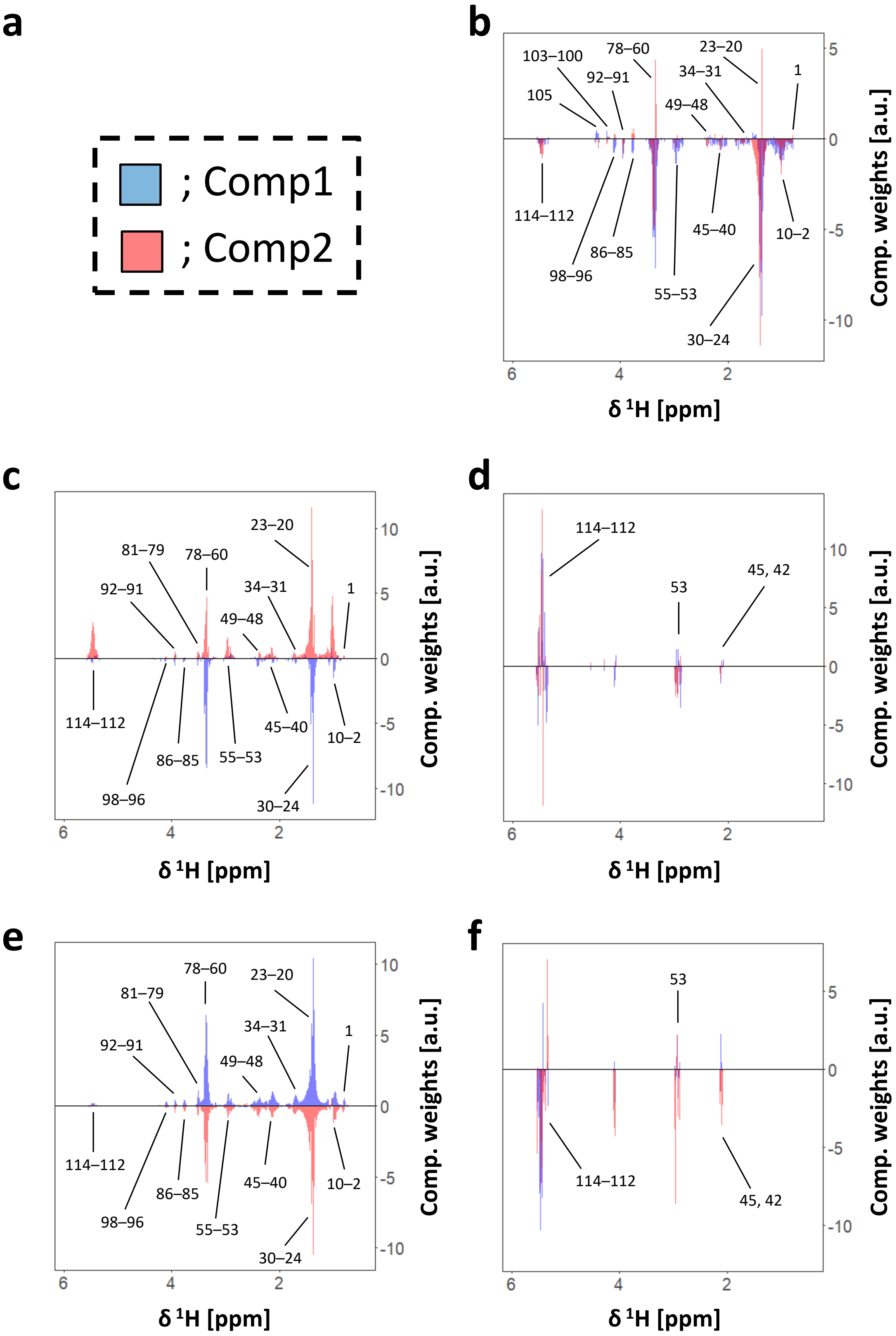

Supplementary Figure 15 Weight plots for Comp.1 and Comp.2 from the 3-way PARAFAC model using pseudo-3D SMOOSY spectra, the second mode loadings B contain feature at each chemical shift. **a** The weights are colored in blue for Comp.1 and red for Comp.2. The weights were calculated using **b** pseudo-3D *D*-SMOOSY, **c** T_2-SMOOSY, **d** $REST_2$-SMOOSY, **e** T_{1}-SMOOSY, and **f** $REST_1$-SMOOSY, respectively.

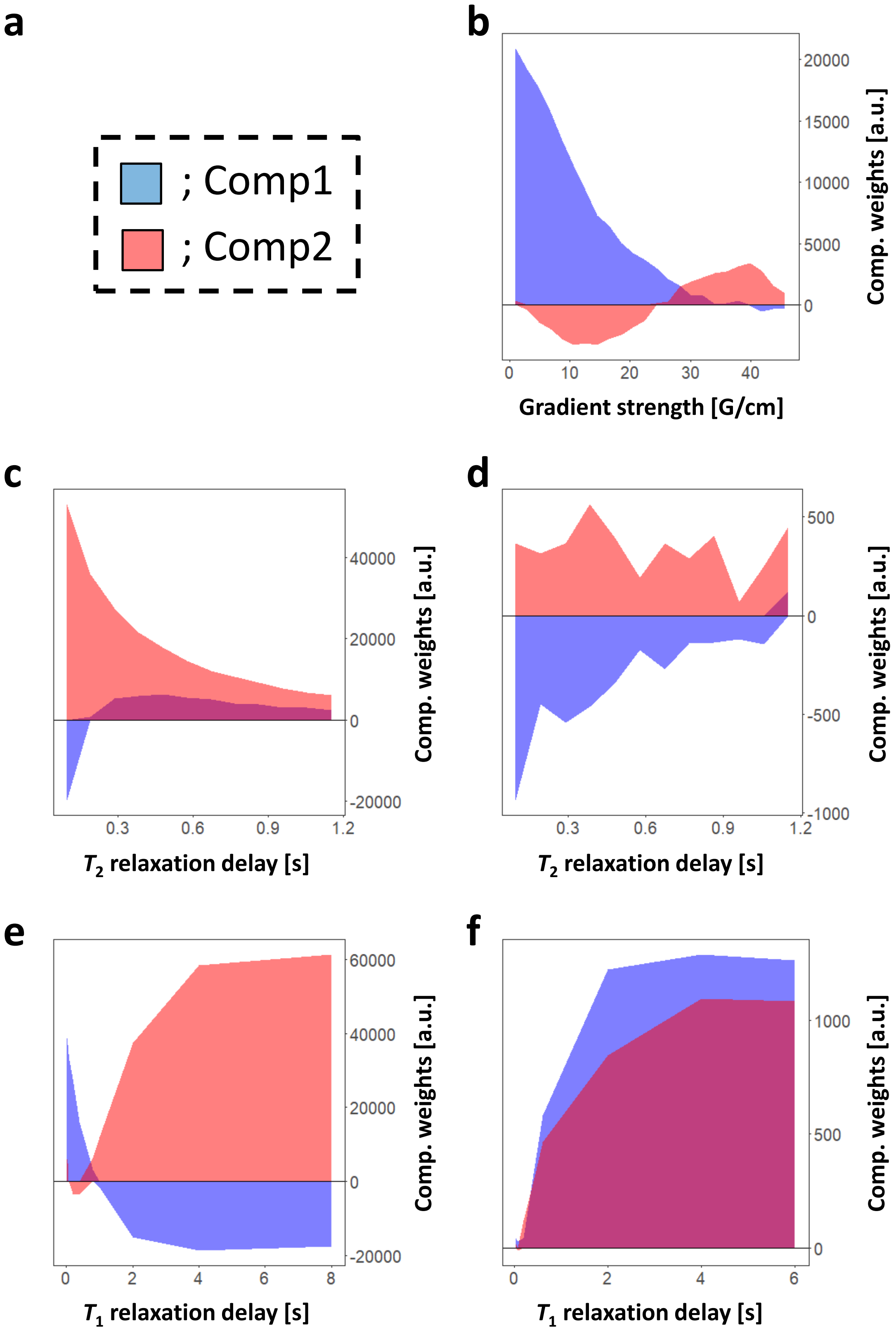

Supplementary Figure 16 Weight plots for Comp.1 and Comp.2 from the 3-way PARAFAC model using pseudo-3D SMOOSY spectra, the third mode loadings C contain feature at each gradient strength or relaxation decay. **a** The weights are colored in blue for Comp.1 and red for Comp.2. The weights were calculated using **b** pseudo-3D *D*-SMOOSY, **c** T_2-SMOOSY, **d** REST$_2$-SMOOSY, **e** T_1-SMOOSY, and **f** REST$_1$-SMOOSY, respectively.

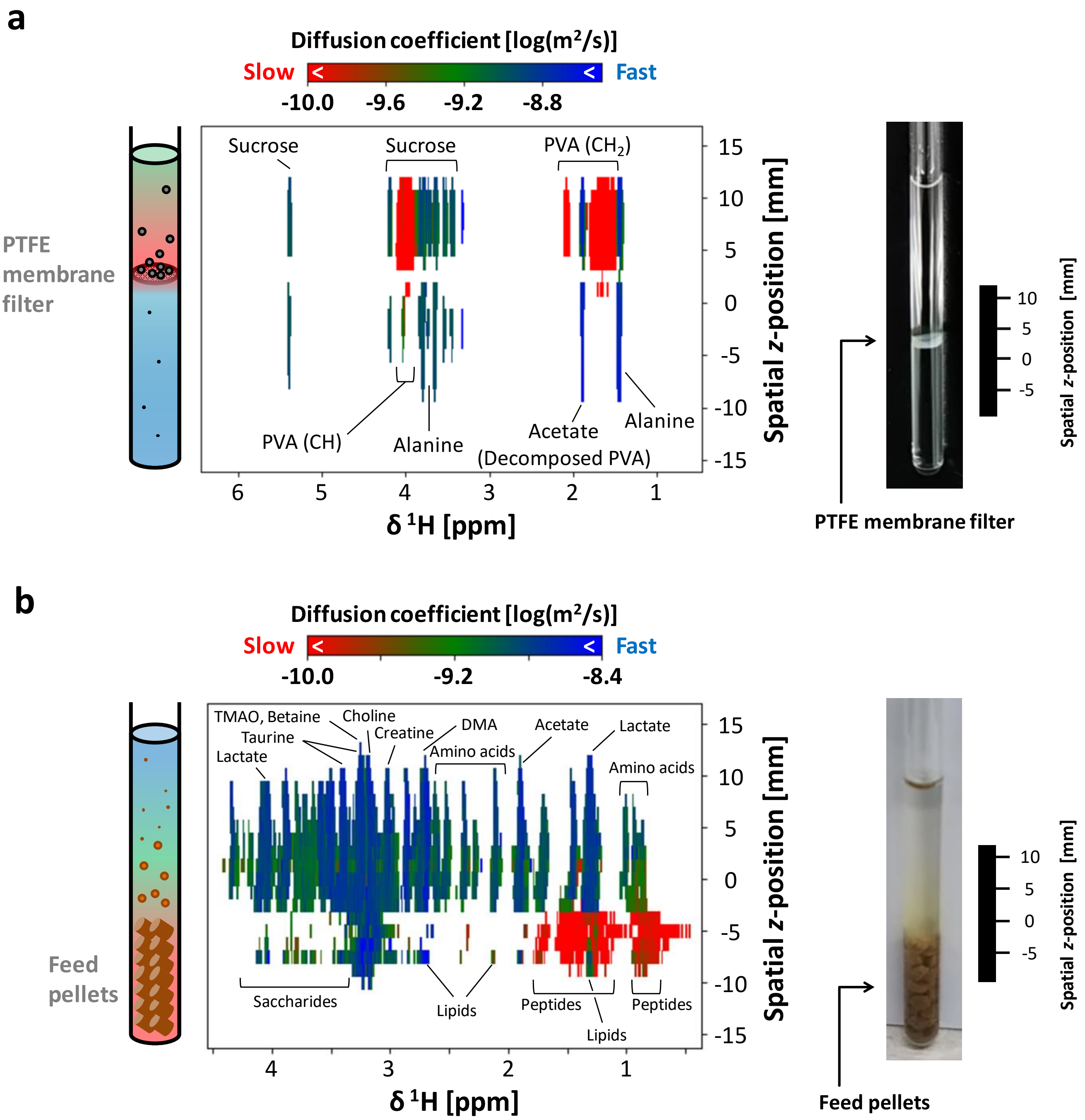

Supplementary Figure 17 Other application examples of *D*-SMOOSY by solution-state NMR without MAS. **a** The pseudo-2D *D*-SMOOSY spectral image of PTFE membrane filtration experiment in 5-mm NMR tube. PVA, alanine, and sucrose are included in D_2O at upside of PTFE membrane filter, the filtered out compounds are included in D_2O at downside of PTFE membrane filter as shown in left sample image. **b** The pseudo-2D *D*-SMOOSY spectral image of feed diffusion experiment in 5-mm NMR tube. Fish feed pellets and D_2O were put into the 5-mm NMR tube, and gradually solved compounds were diffused to upside in NMR tube as shown in left sample image. DMA denotes dimetylamine. The right figures show the pictures of NMR tube after each NMR measurement. The black bars show the range of detected spatial *z*-position. The details of experimental parameters are provided in Supplementary Tables 5 and 6.

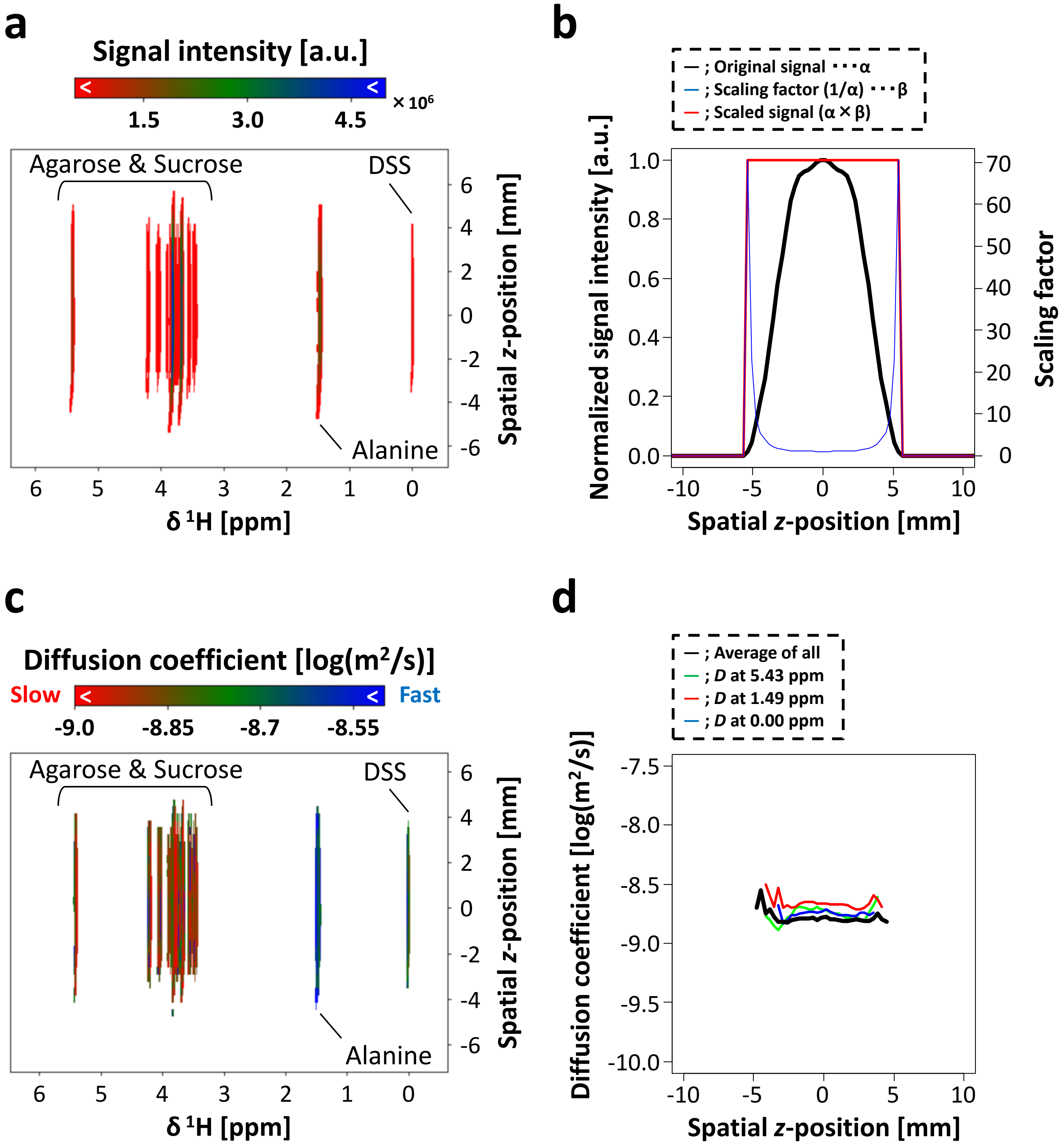

Supplementary Figure 18 a 2D CSI spectrum of homogeneous sample. **b** 2048 columns (dimension of chemical shift) of 2D CSI spectrum were projected for confirming and correcting the distribution of signal intensity at each spatial *z*-position. The signal intensities were normalized that the maximum intensity to be one and minimum intensity to be 0 (black line). Reciprocal numbers of signal intensities at each spatial *z*-position were calculated as scaling factor (blue line). The corrected signals were given by multiplying scaling factor and original signal (red line). Scaling factor calculated in this section was applied for scaling of CSI spectrum of shrimp before PCA (Supplementary Figure 12a, 13a). **c** Pseudo-2D *D*-SMOOSY spectral image of homogeneous sample, was processed from the pseudo-3D SMOOSY spectrum with SMOOSY processor. **d** Diffusion coefficients at each spatial *z*-position were collected from pseudo-2D *D*-SMOOSY spectral image. Average of all diffusion coefficients at each spatial *z*-position were shown by black line. Diffusion coefficients of sucrose (5.43 ppm), alanine (1.49 ppm), and DSS (0ppm) at each spatial *z*-position were also shown by colored lines. These spectra were collected by 3 kHz MAS frequency at 299K. The details of experimental parameters are shown in Supplementary Tables 3 and 4.

Supplementary Table 2 The annotation list for the signals of $^{1}H–^{13}C$ HSQC and CSI of intact shrimp, and for the signals of $^{1}H–^{13}C$ HSQC of extract from powdered shrimp. These spectra are shown in Supplementary Figure 6. The chemical shifts were annotated by a search of the database.

**Peak No.**[a]	**δ ^{1}H [ppm]**	**δ ^{13}C [ppm]**[b]	**Candidate compound**[c]
1	0.79	13.7	Cholesterol
2	0.95	24.7	Cholesterol
3	0.96	16.0	FA (ω -CH_3)
4	1.00	53.2	Cholesterol
5	1.00	16.6	DHA, EPA, FA (n-3) (ω-CH_3)
6	1.02	20.6	Cholesterol
7	1.03	13.7	Isoleucine
8	1.03	23.4	Leucine
9	1.05	24.8	Leucine
10	1.07	59.7	Cholesterol
11	1.07	19.2	Valine
12	1.08	38.9	Cholesterol
13	1.09	21.3	Cholesterol
14	1.11	17.1	Isoleucine
15	1.11	20.7	Valine
16	1.16	39.9	Cholesterol
17	1.19	58.9	Cholesterol
18	1.24	42.1	Cholesterol
19	1.32	43.9	Cholesterol
20	1.35	34.4	FA (ω3-CH_2)
21	1.36	32.0	FA (-CH_2-)n
22	1.38	25.2	FA (ω2-CH_2)
23	1.38	22.8	LA
24	1.45	38.8	Cholesterol
25	1.50	18.8	Alanine
26	1.53	34.7	Cholesterol
27	1.54	33.8	Cholesterol
28	1.59	23.7	Cholesterol
29	1.59	34.5	Cholesterol
30	1.59	30.7	Cholesterol
31	1.67	43.2	Leucine
32	1.68	27.5	FA (β-CH_2)
33	1.76	27.3	Cholesterol
34	1.79	27.1	Leucine, Arginine
35	1.82	43.2	Leucine
36	1.85	33.8	Cholesterol
37	1.91	26.2	GABA
38	1.94	39.9	Cholesterol
39	1.94	30.7	Arginine
40	2.02	34.6	Cholesterol
41	2.07	26.6	Proline
42	2.11	29.7	EPA, FA (olefin-next)
43	2.12	42.3	Cholesterol
44	2.15	29.3	Glutamine
45	2.15	23.0	DHA, EPA, FA (n-3) (ω2-CH_2)
46	2.17	31.6	Proline
47	2.31	44.4	Cholesterol
48	2.39	31.7	Valine, Proline
49	2.39	36.4	FA (α-CH_2)
50	2.46	25.3	DHA (β-CH_2)
51	2.50	34.0	Glutamine
52	2.73	37.1	Asparagine
53	2.92	28.1	DHA, EPA, UFA (olefin-among)
54	3.01	37.2	Asparagine
55	3.04	41.8	Lysine

[a] The peak numbers are the same as those in Supplementary Figure 6. [b] The red colored chemical shifts denote aliased peaks.
[c] Abbreviations: FA, Fatty acid; DHA, Docosahexaenoic acid; EPA, Eicosapentaenoic acid; LA, Linoleic acid; UFA, Unsaturated fatty acid; TMAO, Trimethylamine N-oxide; TAG, Triacylglycerol; GABA, gamma-Aminobutyric acid.

Supplementary Table 2 Continued.

**Peak No.**[a]	**δ^{1}H [ppm]**	**δ^{13}C [ppm]**[b]	**Candidate compound**[c]
56	3.08	39.8	Phenylalanine
57	3.09	31.1	Histidine
58	3.17	77.7	Glucose
59	3.18	50.8	Taurine
60	3.25	43.1	Phosphoethanolamine ($EtNH_2$)
61	3.27	43.3	Arginine
62	3.27	56.1	Choline
63	3.30	30.9	Histidine
64	3.32	56.2	Phosphatidylcholine (Choline NH_3)
65	3.32	79.7	Glucose
66	3.32	62.1	TMAO
67	3.35	73.3	Glucose
68	3.35	38.9	Taurine
69	3.36	48.5	Proline
70	3.36	55.4	Betaine
71	3.37	50.8	MeOH (Methanol)
72	3.39	39.8	Phenylalanine
73	3.39	79.6	Glucose
74	3.39	38.7	Taurine
75	3.40	63.6	Threonine
76	3.45	74.0	Cholesterol
77	3.46	48.4	Proline
78	3.49	63.2	Valine
79	3.49	44.1	Glycine
80	3.54	70.5	Choline
81	3.55	62.3	Isoleucine
82	3.64	53.3	Alanine
83	3.65	57.0	Glutamine
84	3.71	64.4	Glucose
85	3.72	63.1	Dimethyl-Glycine
86	3.74	68.9	Phosphatidylcholine (Choline)
87	3.83	64.1	Glucose
88	3.84	74.5	Glucose
89	3.86	57.8	Histidine
90	3.87	54.5	Asparagine
91	3.91	64.2	Glucose
92	3.92	68.7	Betaine
93	3.98	69.2	Phospholipid (sn-3)
94	4.05	58.7	Choline
95	4.07	71.1	Phospholipid (sn-2)
96	4.08	64.0	Proline
97	4.08	66.2	Phosphatidylcholine (sn-3), Fructose
98	4.10	78.9	Fructose
99	4.13	64.7	Phosphoethanolamine ($EtNH_2$)
100	4.16	67.3	Inosinic acid
101	4.18	68.6	Threonine
102	4.19	67.6	Phospholipid (sn-1)
103	4.20	64.8	(sn-1) TAG
104	4.27	65.2	Phosphatidylcholine (sn-1)
105	4.36	61.9	Phosphatidylcholine (Choline)
106	4.40	64.7	(sn-3) TAG
107	4.46	74.0	Inosinic acid
108	4.52	65.3	Phosphatidylcholine (sn-1)
109	4.75	77.9	Inosinic acid
110	5.33	71.9	(sn-2) TAG
111	5.33	73.4	Phosphatidylcholine (sn-2)
112	5.42	132.3	DHA, EPA, UFA (olefin)
113	5.44	130.7	DHA, EPA, UFA (olefin)
114	5.45	134.2	DHA, EPA, UFA (olefin)

Supplementary Table 3 The acquisition parameters of 2D CSI and pseudo-3D SMOOSY with TopSpin operating system for experiments using intact shrimp and homogeneous samples. The results are shown in Supplementary Figure 9. The pulse programs used in this study are shown in Supplementary Data 1-6.

Acqu. Pars	2D CSI	Pseudo-3D *D*-SMOOSY	Pseudo-3D T_1-SMOOSY	Pseudo-3D T_2-SMOOSY	Pseudo-3D REST$_1$-SMOOSY	Pseudo-3D REST$_2$-SMOOSY
**General**						
Pulse program	ik_CSI	ik_D_SMOOSY	ik_T1_SMOOSY	ik_T2_SMOOSY	ik_REST1_SMOOSY	ik_REST2_SMOOSY
TD (F3)	-	2048	2048	2048	2048	2048
TD (F2)	2048	24	128	128	64	64
TD (F1)	128	128	12	12	8	12
NS	24	16	24	24	64	64
DS	8	4	2	2	4	4
SWH (F3) [Hz]	-	8012.82	8012.82	8012.82	8012.82	8012.82
SWH (F2) [Hz]	8305.647	10002.647	5002.224	5002.224	5002.224	5002.224
SWH (F1) [Hz]	10002.647	5002.225	10002.649	10002.649	10002.647	10002.647
AQ [s]	0.1232896	0.1277952	0.1277952	0.1277952	0.1277952	0.1277952
RG	203	203	203	203	203	203
DW [μs]	60.2	62.4	62.4	62.4	62.4	62.4
DE [μs]	6.5	6.5	6.5	6.5	6.5	6.5
MASR [Hz]	3000	3000	3000	3000	3000	3000
d1 [s]	2	2	2	2	2	2
d2 [s]	0.001	0.001	0.001	0.001	0.001	0.001
d9 [s]	-	-	-	-	0.04	0.04
d16 [s]	-	0.0002	0.0002	0.0002	0.0002	0.0002
d18 [s]	-	-	0.001	0.001	0.001	0.001
d20 [s]	-	0.06	-	0.002	-	0.002
d21 [s]	-	0.005	-	-	-	-
VD [s]	-	-	0.01−8 (12 pt)	0.096−1.152 (12 pt)	0.04−6 (8 pt)	0.096−1.152 (12 pt)
VC [n]	-	-	-	12−144 (12 pt)	-	12−144 (12 pt)
diff2	100%−−100% (128 pt)	100%−−100% (128 pt)	100%−−100% (128 pt)	100%−−100% (128 pt)	100%−−100% (64 pt)	100 %−−100% (64 pt)
GPNAM1	RECT.1	RECT.1	-	-	SMSQ10.100	SMSQ10.100
GPNAM3	-	-	-	-	SMSQ10.100	SMSQ10.100
GPNAM4	-	-	RECT.1	RECT.1	RECT.1	RECT.1
GPNAM5	-	-	SMSQ10.100	-	SMSQ10.100	-
GPNAM6	-	SMSQ10.100	-	-	-	-
GPNAM7	-	SMSQ10.100	-	-	-	-
GPNAM8	-	SMSQ10.100	SMSQ10.100	SMSQ10.100	SMSQ10.100	SMSQ10.100
GPZ1 [%]	20	20	-	-	23	23
GPZ3 [%]					40	40
GPZ4 [%]	-	-	20	20	10	10
GPZ5 [%]	-	-	10	-	13	-
GPZ6 [%]	-	100	-	-	-	-
GPZ7 [%]	-	-17.13	-	-	-	-
GPZ8 [%]	-	-13.17	13	13	13	13
p16 [us]	200	200	1000	-	1000	1000
p18 [us]	-	-	1000	1000	1000	1000
p19 [us]	-	600	200	200	200	200
p30 [us]	-	1800	-	-	-	-
**Channel f1**						
NUC1	1H	1H	1H	1H	1H	1H
P1 [μs]	10	10	10	10	10.15	10.15
P6 [us]	-	-	-	-	26	26
P8 [us]	-	-	1000	1000	1000	1000
P12 [us]	-	-	-	-	7752	7752
P17 [us]	-	-	-	-	2500	2500
O1 [Hz]	2464.83	2464.83	2464.83	2464.83	2456.53	2456.53
PLW0 [W]	-	-	0	0	0	0
PLW1 [W]	5.5	5.5	5.5	5.5	5.5	5.5
PLW9 [W]	4.40E-05	4.40E-05	4.40E-05	4.40E-05	4.40E-05	4.40E-05
PLW10 [W]	-	-	-	-	9.27E-01	9.27E-01
SFO1 [MHz]	500.1324648	500.1324648	500.1324648	500.1324648	500.1324565	500.1324565
SPNAM6	-	-	Squa100.1000	Squa100.1000	Squa100.1000	Squa100.1000
SPOAL6	-	-	0.5	0.5	0.5	0.5
SPOFFS6 [Hz]	-	-	0	0	0	0
SPW6 [W]	-	-	0.00062661	0.00062661	0.00062661	0.00062661
SPNAM12	-	-	-	-	Reburp.1000	Reburp.1000
SPOAL12	-	-	-	-	0.5	0.5
SPOFFS12 [Hz]	-	-	-	-	169.75	169.75
SPW12 [W]	-	-	-	-	0.0059156	0.0059156
CNST18	-	-	85	-	85	-
CNST29	-	-	4.6869998	4.6869998	4.9120002	4.9120002
Experimental time	58 min.	16 hr. 23 min.	1 day 12 hr. 24 min.	1 day 4 hr. 36 min.	1 day 2 hr. 10 min.	1 day 1 hr. 6 min.

Supplementary Table 4 The processing parameters of 2D CSI and pseudo-3D SMOOSY with TopSpin operating system for experiments using intact shrimp and homogeneous samples. The results are shown in Supplementary Figure 9. The pulse programs used in this study are shown in Supplementary Data 1-6.

**Proc. Pars**	**2D CSI**	**Pseudo-3D *D*-SMOOSY**	**Pseudo-3D T_1-SMOOSY**	**Pseudo-3D T_2-SMOOSY**	**Pseudo-3D $REST_1$-SMOOSY**	**Pseudo-3D $REST_2$-SMOOSY**
SI (F3)	-	2048	2048	2048	2048	2048
SI (F2)	2048	32	128	128	64	64
SI (F1)	128	128	16	16	8	16
WDW (F3)	-	SINE	SINE	SINE	SINE	SINE
WDW (F2)	SINE	no	SINE	SINE	SINE	SINE
WDW (F1)	SINE	SINE	no	no	no	no
SSB (F3)	-	1	1	1	1	1
SSB (F2)	1	0	0	0	0	0
SSB (F1)	0	0	0	0	0	0
PH_mod (F3)	-	pk	pk	pk	pk	pk
PH_mod (F2)	pk	mc	mc	mc	mc	mc
PH_mod (F1)	mc	mc	mc	mc	mc	mc
ABSG (F3)	-	5	5	5	5	5
ABSG (F2)	5	5	1	1	1	1
ABSG (F1)	1	1	5	5	5	5
BC_mod (F3)	-	quad	qfil	quad	quad	quad
BC_mod (F2)	quad	no	quad	quad	quad	quad
BC_mod (F1)	quad	quad	no	no	no	no
ME_mod (F3)	-	no	no	no	no	no
ME_mod (F2)	no	no	LPfc	LPfc	LPfc	LPfc
ME_mod (F1)	LPfc	LPfc	no	no	no	no
NCOEF (F3)	-	0	0	0	0	0
NCOEF (F2)	0	0	8	8	8	8
NCOEF (F1)	8	8	0	0	0	0
REVERSE (F3)	-	FALSE	FALSE	FALSE	FALSE	FALSE
REVERSE (F2)	FALSE	FALSE	TRUE	TRUE	TRUE	TRUE
REVERSE (F1)	TRUE	TRUE	FALSE	FALSE	FALSE	FALSE

Supplementary Table 5 Acquisition parameters for pseudo-3D SMOOSY with TopSpin operating system for PTFE membrane filtration and feed diffusion/dissolution experiments. Results are shown in Fig. 4 and Supplementary Figure 17. Pulse programs used in this study are shown in Supplementary Data 1-6.

Acqu. Pars	Pseudo-3D *D*-SMOOSY[a]	Pseudo-3D *D*-SMOOSY[b]	Pseudo-3D T_2-SMOOSY[c]
**General**			
Pulse program	ik_D_SMOOSY	ik_D_SMOOSY	ik_T2_SMOOSY
TD (F3)	2048	2048	1024
TD (F2)	14	14	16
TD (F1)	32	32	6
NS	16	16	2
DS	4	4	2
SWH (F3) [Hz]	8196.722	8196.722	11363.637
SWH (F2) [Hz]	10002.647	10002.647	7002.793
SWH (F1) [Hz]	7002.793	7002.793	14003.065
AQ [s]	0.124928	0.124928	0.045056
RG	101	101	101
DW [μs]	61	61	44
DE [μs]	10	10	10
MASR [Hz]	-	-	-
d1 [s]	1	1	0.6
d2 [s]	0.001	0.001	0.001
d9 [s]	-	-	-
d16 [s]	0.0002	0.0002	0.0002
d18 [s]	-	-	0.001
d20 [s]	0.06	0.06	0.002
d21 [s]	0.005	0.005	-
VD [s]	-	-	0.2−1.2 (6 pt)
VC [n]	-	-	25−150 (6 pt)
diff2	100%−−100% (32 pt)	100%−−100% (32 pt)	100%−−100% (16 pt)
GPNAM1	RECT.1	RECT.1	-
GPNAM3	-	-	-
GPNAM4	-	-	RECT.1
GPNAM5	-	-	-
GPNAM6	SMSQ10.100	SMSQ10.100	-
GPNAM7	SMSQ10.100	SMSQ10.100	-
GPNAM8	SMSQ10.100	SMSQ10.100	SMSQ10.100
GPZ1 [%]	5	5	-
GPZ3 [%]	−	−	−
GPZ4 [%]	-	-	2.5
GPZ5 [%]	-	-	-
GPZ6 [%]	100	100	-
GPZ7 [%]	-17.13	-17.13	-
GPZ8 [%]	-13.17	-13.17	13
p16 [us]	200	200	-
p18 [us]	-	-	1000
p19 [us]	600	600	200
p30 [us]	1800	1800	-
**Channel f1**			
NUC1	1H	1H	1H
P1 [μs]	11	11.75	11.8
P6 [us]	-	-	-
P8 [us]	-	-	1000
P12 [us]	-	-	-
P17 [us]	-	-	-
O1 [Hz]	3287.31	3290.71	3293.25
PLW0 [W]	-	-	0
PLW1 [W]	11.194	11.194	11.188
PLW9 [W]	9.86E-05	9.86E-05	2.86E-05
PLW10 [W]	-	-	-
SFO1 [MHz]	700.1532873	700.1532907	700.1532933
SPNAM6	-	-	Squa100.1000
SPOAL6	-	-	0.5
SPOFFS6 [Hz]	-	-	0
SPW6 [W]	-	-	0.0016749
SPNAM12	-	-	-
SPOAL12	-	-	-
SPOFFS12 [Hz]	-	-	-
SPW12 [W]	-	-	-
CNST18	-	-	-
CNST29	-	-	4.704
Experimental time	6 hr. 22 min.	6 hr. 22 min.	5 min

[a] This *D*-SMOOSY experiment was performed for PTFE membrane filtration. The processed spectral image is shown in Supplementary Figure 17a.
[b] This *D*-SMOOSY experiment was performed for feed diffusion. The processed spectral image is shown in Supplementary Figure 17b.
[c] This T_2-SMOOSY experiment was performed for feed dissolution. The processed spectral image is shown in Fig. 4.

Supplementary Table 6 Processing parameters for pseudo-3D SMOOSY with TopSpin operating system for PTFE membrane filtration and feed diffusion/dissolution experiments. Results are shown in Fig. 4 and Supplementary Figure 17. Pulse programs used in this study are shown in Supplementary Data 1-6.

Proc. Pars	Pseudo-3D *D*-SMOOSY[a]	Pseudo-3D *D*-SMOOSY[b]	Pseudo-3D T_2-SMOOSY[c]
SI (F3)	2048	2048	2048
SI (F2)	16	16	128
SI (F1)	32	32	8
WDW (F3)	SINE	SINE	SINE
WDW (F2)	no	no	SINE
WDW (F1)	SINE	SINE	no
SSB (F3)	1	1	1
SSB (F2)	0	0	0
SSB (F1)	0	0	0
PH_mod (F3)	pk	pk	pk
PH_mod (F2)	mc	mc	mc
PH_mod (F1)	mc	mc	mc
ABSG (F3)	5	5	5
ABSG (F2)	5	5	1
ABSG (F1)	1	1	5
BC_mod (F3)	quad	quad	quad
BC_mod (F2)	no	no	quad
BC_mod (F1)	quad	quad	no
ME_mod (F3)	no	no	no
ME_mod (F2)	no	no	no
ME_mod (F1)	LPfc	LPfc	no
NCOEF (F3)	0	0	0
NCOEF (F2)	0	0	0
NCOEF (F1)	8	8	0
REVERSE (F3)	FALSE	FALSE	FALSE
REVERSE (F2)	FALSE	FALSE	TRUE
REVERSE (F1)	TRUE	TRUE	FALSE

[a] This *D*-SMOOSY experiment was performed for PTFE membrane filtration. The processed spectral image is shown in Supplementary Figure 17a.
[b] This *D*-SMOOSY experiment was performed for feed diffusion. The processed spectral image is shown in Supplementary Figure 17b.
.[c] This T_2-SMOOSY experiment was performed for feed dissolution. The processed spectral image is shown in Fig. 4.

Supplementary References

1. Helmus, J. J. & Jaroniec, C. P. Nmrglue: an open source Python package for the analysis of multidimensional NMR data. *J. Biomol. NMR* **55**, 355−367 (2013).
2. Moré, J. J. The Levenberg-Marquardt algorithm: implementation and theory. *Numerical Anal.* **630**, 105–116 (1978).
3. Cohen, Y. & Slovak, S. Diffusion NMR for the characterization, in solution, of supramolecular systems based on calixarenes, resorcinarenes, and other macrocyclic arenes. *Org. Chem. Front.* **6**, 1705−1718 (2019).
4. Wisniewska, M. A. & Seland, J. G. Investigating structure-dependent diffusion in hydrogels using spatially resolved NMR spectroscopy. *J. Colloid Interface Sci.* **533**, 671−677 (2019).
5. Savorani, F., Tomasi, G. & Engelsen, S. B. icoshift: A versatile tool for the rapid alignment of 1D NMR spectra. *J. Magn. Reson.* **202**, 190−202 (2010).
6. van Rossum, B. J., Förster, H. & de Groot, H. J. M. High-field and high speed CP-MAS ^{13}C NMR heteronuclear dipolar-correlation spectroscopy of solids with frequency-switched Lee–Goldburg homonuclear decoupling. *J. Magn. Reson.* **124**, 516−519 (1997).
7. Yamada, S. et al. InterSpin: integrated supportive webtools for low- and high-field NMR analyses toward molecular complexity. *ACS Omega* **4**, 3361−3369 (2019).
8. Kang, X. et al. Molecular architecture of fungal cell walls revealed by solid-state NMR. *Nat. Commun.* **9**, 2747 (2018).
9. Kono, H. Two-dimensional magic angle spinning NMR investigation of naturally occurring chitins: Precise ^{1}H and ^{13}C resonance assignment of α- and β-chitin. *Biopolymers* **75**, 255−263 (2004).
10. Mobarhan, Y. L. et al. Comprehensive multiphase NMR applied to a living organism. *Chem. Sci.* **7**, 4856−4866 (2016).
11. Matsui, S. Solid-state NMR imaging by magic sandwich echoes. *Chem. Phys. Lett.* **179**, 187−190 (1991).
12. Schock, T. B. et al. Evaluation of Pacific white shrimp (Litopenaeus vannamei) health during a superintensive aquaculture growout using NMR-based metabolomics. *PLoS One* **8**, e59521 (2013).
13. Zerbst-Boroffka, I., Kamaltynow, R. M., Harjes, S., Kinne-Saffran, E. & Gross, J. TMAO and other organic osmolytes in the muscles of amphipods (Crustacea) from shallow and deep water of Lake Baikal. *Comp. Biochem. Physiol., Part A: Mol Integr. Physiol.* **142**, 58−64 (2005).
14. Fuzessery, Z. M. & Childress, J. J. Comparative chemosensitivity to amino acids and their role in the feeding activity of bathypelagic and littoral crustaceans. *Biol. Bull.* **149**, 522−538 (1975).
15. Hogben, H. J., Krzystyniak, M., Charnock, G. T. P., Hore, P. J. & Kuprov, I. Spinach – A software library for simulation of spin dynamics in large spin systems. *J. Magn. Reson.* **208**, 179−194 (2011).
16. Pegg, D. T., Bendall, M. R. & Doddrell, D. M. Randomization of spins in heteronuclear pulse sequences. *J. Magn. Reson.* **49**, 32−47 (1982).
17. Dal Poggetto, G., Castañar, L., Adams, R. W., Morris, G. A. & Nilsson, M. Relaxation-encoded NMR experiments for mixture analysis: REST and beer. *Chem. Commun.* **53**, 7461−7464 (2017).
