# Supplementary material for: Spatial molecular-dynamically ordered NMR spectroscopy of intact bodies and heterogeneous systems
Source: Commun Chem. 2020 Jun 26;3:80. doi: 10.1038/s42004-020-0330-1 (PMC9814264; doi:10.1038/s42004-020-0330-1)
Supplement: Supplementary file 3 — Description of Additional Supplementary Files [file 42004_2020_330_MOESM3_ESM.pdf]

Description of Additional Supplementary Files

File Name: Supplementary Data 1
Description: Pulse program of 2D CSI for Bruker Avance II/III/NEO

File Name: Supplementary Data 2
Description: Pulse program of pseudo-3D D-SMOOSY for Bruker Avance II/III/NEO

File Name: Supplementary Data 3
Description: Pulse program of pseudo-3D T1-SMOOSY for Bruker Avance II/III/NEO

File Name: Supplementary Data 4
Description: Pulse program of pseudo-3D T2-SMOOSY for Bruker Avance II/III/NEO

File Name: Supplementary Data 5
Description: Pulse program of pseudo-3D REST1-SMOOSY for Bruker Avance II/III/NEO

File Name: Supplementary Data 6
Description: Pulse program of pseudo-3D REST2-SMOOSY for Bruker Avance II/III/NEO
